# Organic/Organic Heterointerface Engineering to Boost Carrier Injection in OLEDs

**DOI:** 10.1038/srep42787

**Published:** 2017-02-20

**Authors:** Mohammadreza Fathollahi, Mohsen Ameri, Ezeddin Mohajerani, Ebrahim Mehrparvar, Mohammadrasoul Babaei

**Affiliations:** 1 Laser and Plasma Research Institute, Shahid Beheshti University, G.C., Tehran 1983963113, Iran; 2 Department of Physics, Bu-Ali Sina University, P.O. Box 65174, Hamedan, Iran

## Abstract

We investigate dynamic formation of nanosheet charge accumulations by heterointerface engineering in double injection layer (DIL) based organic light emitting diodes (OLEDs). Our experimental results show that the device performance is considerably improved for the DIL device as the result of heterointerface injection layer (HIIL) formation, in comparison to reference devices, namely, the current density is doubled and even quadrupled and the turn-on voltage is favorably halved, to 3.7 V, which is promising for simple small-molecule OLEDs. The simulation reveals the (i) formation of dynamic p-type doping (DPD) region which treats the quasi Fermi level at the organic/electrode interface, and (ii) formation of dynamic dipole layer (DDL) and the associated electric field at the organic/organic interface which accelerates the ejection of the carriers and their transference to the successive layer. HIIL formation proposes alternate scenarios for device design. For instance, no prerequisite for plasma treatment of transparent anode electrode, our freedom in varying the thicknesses of the organic layers between 10 nm and 60 nm for the first layer and between 6 nm and 24 nm for the second layer. The implications of the present work give insight into the dynamic phenomena in OLEDs and facilitates the development of their inexpensive fabrication for lighting applications.

Material interfaces, intermediate layers, and charge transfer across the interfaces play determinant roles in all aspect of nanoscale systems from organic electronic devices to nanobio systems^1^,^2^,^3^. Energy barrier at the interface between the metal electrode and the organic layer in organic light emitting diodes (OLEDs) prevents the effective carrier injection into the active layer and consequently results in unbalanced electron-hole currents flowing through the device^4^,^5^,^6^. Historically, employment of organic hole-injection buffer, namely copper phthalocyanine (CuPc), was demonstrated to improve the device performance^7^,^8^,^9^,^10^. Utilization of poly(3,4-ethylenedi-oxythiophene):poly(styrenesulfonate) (PEDOT:PSS) as hole injection layer has gained popularity due to excellent properties such as ease of deposition, reduction of shorts, and low-cost of production^11^,^12^,^13^,^14^,^15^. Also, few-nanometer-thick layer of metal oxides and inorganic salts such as MoO_3_ and LiF have proven their effectiveness in carrier injection/extraction enhancement^16^,^17^,^18^,^19^,^20^,^21^. Of upmost importance, molecular n-type/p-type doping of organic materials has provided a breakthrough concept to tackle the charge balancing issue in OLED fabrication^22^,^23^,^24^. In addition, some innovative concepts have been recently proposed to enhance the carrier injection; for example, nanocomposition of organic and inorganic materials^25^,^26^, multiple quantum well structure^27^,^28^,^29^,^30^, nanoscale plasmonic and geometrical field enhancement^31^,^32^,^33^, and interestingly, mobile ion dynamic doping (DD) in light-emitting electrochemical cells^34^,^35^,^36^.

Effective enhancement of carrier injection at metal/organic interface is fundamentally challenging. For example, for n-type-doping systems, the highest-occupied-molecular orbital (HOMO) of the dopant should lie above the lowest-unoccupied-molecular orbital (LUMO) of the matrix *i.e.* the dopant should possesses LUMO energy level close to the vacuum level which makes the dopant highly unstable against the oxidation^37^. Therefore, the underlying chemistry for synthesis of stable dopant dramatically increases the price of the final product^38^,^39^,^40^,^41^. On the other hand, the performance of the devices with single injection layer is generally low and therefore properly engineered structures are required for commercial applications^42^,^43^,^44^,^45^,^46^. Also, employment of compositional and multipart layer structure as carrier injection layer in organic devices requires further investigation in order to understand the operational principle and crucial parameters^47^,^48^,^49^,^50^. In particular, spatial mapping of charge distribution and emission profile within the device^51^,^52^,^53^, charge injection and impedance spectroscopy, and computer simulation can be used to understand the nature of the operating process^54^,^55^,^56^,^57^. Nonetheless, the device simulation has been proven to require complicated approaches such as multi-scale and molecular-scale modeling, which are generally not suitable for benchmarking the device fabrication optimization^55^,^58^,^59^,^60^. Furthermore, dynamic phenomena particularly dynamic doping and dynamic dipole layer (DDL) have attracted little attention so far^34^. By dynamic phenomena, we mean the effects which take place as the bias voltage is applied and disappear when the power supply is switched off (Fig. 1).

In the present report, we demonstrate the formation of dynamic nanosheet carrier accumulations in double injection layer (DIL), forming a heterointerface injection layer (HIIL), can strikingly improve the charge balancing in OLEDs. Therefore, the carrier injection issue can be tackled by device physics rather than material chemistry through employing a organic/organic heterointerface as hole injection layer. Our experimental measurements of the fabricated devices reveal that the turn-on voltage halved for the HIIL device, whilst its current density is doubled or even fourfold compared to reference devices. Afterwards, the experimental results are combined with computational modeling to unravel the underlying physics. Based on simulation results, two dynamic phenomena are observed, firstly, the DD at the organic/electrode interface and secondly the dynamic dipole layer at the organic/organic interface. The former can cause hole Fermi level (E_F_) lowering, and the latter can favorably impose an electric field as driving force to transfer the carriers to the adjacent layer in similar to coulomb field establishment due to charge accumulation in high gain organic photodetectors (Fig. 2). Additionally, our model differs from drift-diffusion framework by accounting for the carrier localized states in organic materials, and results in quantitative layer-by-layer optimization in contrast to Monte Carlo method which accounts merely for the details of the molecular-scale phenomena. Finally, by means of computational design, we investigate various preparation scenarios for the proposed heterointerface injection layer to obtain the optimized fabrication recipes and also discuss the benefits of the heterointerface injection layer.

## Experimental

### Materials

Poly(3,4-ethylenedi-oxythiophene):poly(styrenesulfonate) (PEDOT:PSS) conducting polymer and Copper(II) phthalocyanine (CuPc) small molecule were employed alone or in adjacent to form the hole injection layer and to examine the concept of dynamic functionalizing of the device structure. Tris(8-hydroxyquinolinato) aluminum (Alq_3_) and N,N’-bis(3-methylphenyl)-N,N’-bis(phenyl)-benzidine (TPD) were used as active layer and hole transport layer, respectively. Also, indium tin oxide (ITO) coated substrate was exploited as the anode electrode and Ca was deposited as an efficient cathode electrode. The thin layer of calcium was covered by Ag layer for protection and electrical connection to the outer circuit. All materials were purchased from Sigma-Aldrich and employed without further purification.

### Single injection layer (SIL) and double injection layer (DIL) based OLEDs fabrication and characterization

**Figure 1:**
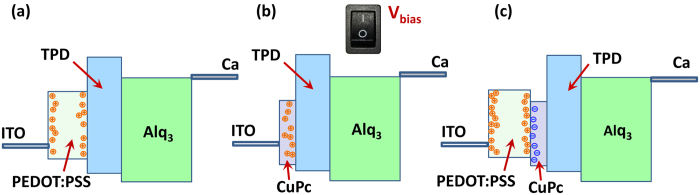
Comparison of fabricated reference devices with SIL (**a** and **b**) and with HIIL (**c**). As the bias voltage is applied and the carriers begin to be injected and transported, three nanosheets of charge carrier accumulation are formed within the HIIL to further enhancement of hole injection.

Patterned ITO coated substrates were firstly cleaned in ultrasonic bath in detergent, acetone, dichloromethane, methanol, isopropanol, and deionized water each for 20 minutes. Subsequently, 5 sets of three devices with different hole injection layer were prepared. PEDOT:PSS solution was diluted slightly and then spin-coated at 3000 rpm for 30 seconds and finally backed at 120 °C to form the hole injection layer in the first device. The thickness of the layer was ~40 nm. CuPc was thermally evaporated to form the hole injection layer in the second device. The thickness of the layer was ~15 nm. For the third OLED with double injection layer, CuPc layer was deposited in adjacent to PEDOT:PSS layer to form the double hole injection layer. Afterwards, thermal deposition of TPD as hole transport layer with thickness of ~35 nm and Alq_3_ as active layer with thickness of ~65 nm were performed. Finally, Ca/Ag cathode electrode was evaporated on the top of the organic layers through a shadow mask in the base pressure of ~10^−5^ mbar. The thickness of Ca and Ag layers were 30 nm and 90 nm, respectively. Figure 1 presents the structure of the fabricated devices. Then, the fabricated devices were characterized. The current density-voltage (*J-V*) curves were measured by Keithley 2400 source-measurement unit. The emission intensity against voltage relations (*L-V*) of the devices were recorded by Mastech-MS6612 optical instrument. The thickness of the layers was quantified by Dektak8000 profilemeter. Finally, the electroluminescence (*EL*) spectra were collected by USB2000 Ocean Optics instrument.

### Simulation model

A customized version of numerical model, called MOLED, was employed to simulate the characteristics of the fabricated devices^47^,^61^ (Supporting information). The numerical model solves four equations self-consistently to obtain the dynamic of the carriers^47^,^61^,^62^. The carrier transition between the electrode and the organic molecules was formulated by Bardeen tunneling theory and basically accounts for the localized nature of the electronics states in the organic solids^61^,^63^,^64^:


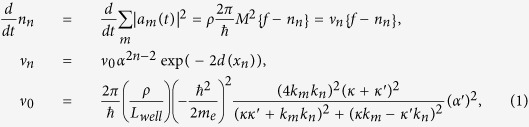


where *n*_*n*_ is the charge density, *f* the Fermi-Dirac function, *ν*_*n*_ the hopping rate, *M* the matrix element, *ħ* the reduced Planck’s constant, and *ρ* the metal density of states per unit length. The *α* shows carrier wave’s amplitude attenuation caused by passing through the narrow barriers in the organic materials and the term *exp*(*−2d*(*x*_*n*_)) includes the effect of slow variation in potential profile. The term *ν*_*0*_ has the unit of *s*^*−*1^ and express the hopping rate between metal and organic molecules, in which *L*_*well*_ is the carrier delocalization length in the organic semiconductor, *m*_*e*_ the electron mass, *α*′ the wave function attenuation at the contact caused by the barrier, and finally *K*_*m*_, *K*_*n*_, *κ*, and *κ*′ are the carrier wave vectors in different regions of the metal-organic contact.

The carrier transition among the organic molecules with localized states was modeled by the master equation as follows^47^,^61^,^62^,^64^:


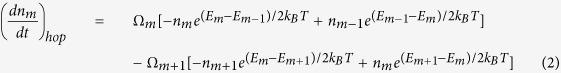


where *Ω*_*i*_ is the hopping frequency among organic molecules, *E*_*i*_ the energy level of the organic site i, *k*_*B*_ the Boltzmann’s factor, and T the temperature. The hopping frequency can be adapted from mobility measurements as follows^61^,^62^,^64^:


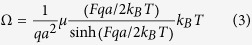


where *a* is the distance between carrier sites (organic molecules), *F* the external electric field, and *q* the elementary carrier charge. The recombination process within the emissive layer was described by Langevin relation. The shift in the molecular energy levels during the device operation was termed by considering the image force potential, the discreteness within the first monolayers, and the space charges of the organic layers and the electrodes. The values of the main parameters for modeling; namely, the energy levels and the mobility of the materials are presented in Table 1. Several microscopic quantities and different device characteristics were calculated namely electron and hole densities, shifts in the molecular energy levels, local electric filed, different components of the current flow, contribution to the electron-hole recombination, total current density, total emitted light, and different device efficiencies for the given bias voltages.

## Results and Discussion

### Characteristic improvement

Figure 3a presents the improvement in the *J-V* characteristics of the device with PEDOT:PSS/CuPc heterointerface as the injection layer with respect to the reference devices with PEDOT:PSS and CuPc single injection layer (SIL). As can be seen, the current density increases from ~64 mAcm^−2^ for PEDOT device and ~120 mAcm^−2^ for CuPc device to ~240 mAcm^−2^ for DIL PEDOT-CuPc device at the bias voltage of 10 V. Also, Fig. 3b compares the emission intensity for three fabricated OLEDS with different injection layers. As seen, the emission intensity increases for the device which employs the heterointerface injection layer in proportional to the current density. Interestingly, the turn-on voltage from 8 V for PEDOT:PSS device and 6 V for CuPc device is reduced to value of 3.7 V for the device with PEDOT:PSS/CuPc injection layer. This onset voltage approaches the thermodynamic limit of 2.7 V, as attributed to Alq_3_ band gap, and is considerable for simple small-molecule organic LED without n-type/p-type molecular doped injection layers. Figure 3c presents the EL spectra of the fabricated devices at the bias voltage of 10 V. Again, the enhancement in the emission intensity can be observed for PEDOT:PSS/CuPC HIIL device in comparison to the SIL devices.

### Interpretation of devices performance enhancement by computational modeling analysis

Device simulation can give insight into the dynamic of the carrier within the organic layers and consequently reveals the underlying physics of the operating device. Figure 4 compares the result of modeling with experimental data in logarithmic scale. Figure 5 also presents the results of numerical modeling for *J-V* and *L-V* characteristics of the three fabricated devices in linear scale. As can be seen, the improvement in device characteristics is replicated in simulation results.

**Figure 2:**
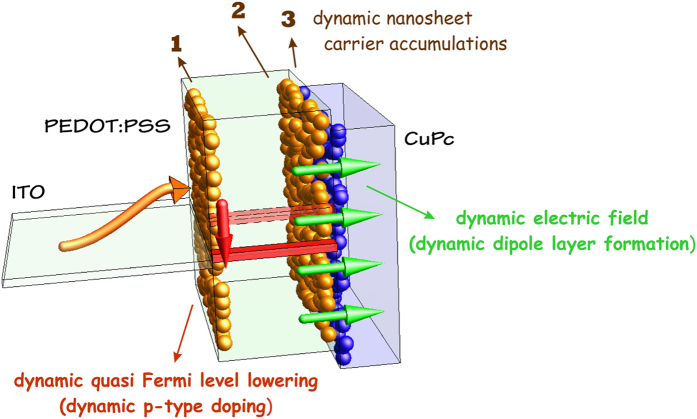
The formation of the HIIL. The dynamic formation of nanosheet charge accumulations causes DPD and DDL formation. The former can cause hole E_F_ lowering, and the latter can favorably impose a driving force to transfer the carriers to the adjacent layer. The presented structure can be considered as an example for functionalizing the device structure.

As mentioned earlier, the turn-on voltage of the OLEDS reduces from 8 V and 6 V for SIL devices to 3.7 V for the HIIL device. Figure 6a,c and e compare the charged carrier distributions within and at the interface of the organic layers for all the devices at the bias voltage of 4.0 V in which PEDOT:PSS/CuPc device has already turned on but the other two devices have not. It can be observed that utilization of injection layer leads in dynamic formation of nanosheet charge accumulations at the metal-organic interface. Figure 2 depicts the formation of the charge accumulations in PEDOT:PSS/CuPc device after the application of the external bias voltage. This nanosheet charge accumulations shift the so-called quasi E_F_ and can be interpreted as dynamic doping effect in the organic material.

Stimulatingly, another dynamic effect takes place by supplement of the second injection layer. As depicted in Fig. 6e, two nanosheets of opposite charges are accumulated at the organic/organic interface and consequently a dynamic electric field is established within the heterointerface injection layer which ejects the carriers from the electrode and passes them to the successive layer. Recently, formation of dynamic Coulomb field has been reported due to the charge accumulation in nanocomposite organic photodetectors which leads in remarkably large carrier injection during light illumination. In contrast to the quantum well here which is formed due to the stacking of three organic layers, the trap states in nanocomposite due to energy level mismatch between donor and acceptor species are responsible for charge accumulation and the dynamic electric field formation^65^,^66^,^67^,^68^.

The origin of nanosheet charge accumulations and therefore the formation of the dipole layer can be revealed from the energy level diagram as depicted in Fig. 6b,d and f. In particular, the proper combination of PEDOT:PSS, CuPc, and TPD layers in the device structure forms adequate potential wells for the both carriers, consequently the electrons and the holes become accumulated and a charge dipole layer arises (Fig. 1c). Figure 7a,b demonstrate the carrier profiles and the energy levels for the double injection device at the operating voltage of 10 V. Evidently, it can be seen the dynamical phenomena remain and even amplified at higher bias voltages. Figure 2 depicts the formation of the nanosheet charge accumulations and consequent DD at the metal-organic interface (additional charge carriers) and dynamic diploe layer and its associated electric field at the organic-organic interface.

In a simpler case, barrier reduction is also reported by employing thin CuPc film in a SIL OLED^69^. Similar behavior by formation of p- and n-doped regions adjacent to the electrodes is reported to enhance the carrier injection in light-emitting electrochemical cells in which the redistribution of mobile ions allows for the growth of the doped regions^70^,^71^. Less efficient method of application of two transport layers doped by MoO_3_ also led to a drop in driving voltage which cannot be assigned to DDL formation as explored here^72^,^73^. Enhancement in hole injection could also arise from organic multi-quantum well OLED with further complexity^28^. In contrast, herein, this promising concept is extended by employing heterointerface injection layer. In other words, three charge carrier accumulations in form of nanosheets join forces to enhance carrier injection at the metal-organic interface.

**Figure 3:**
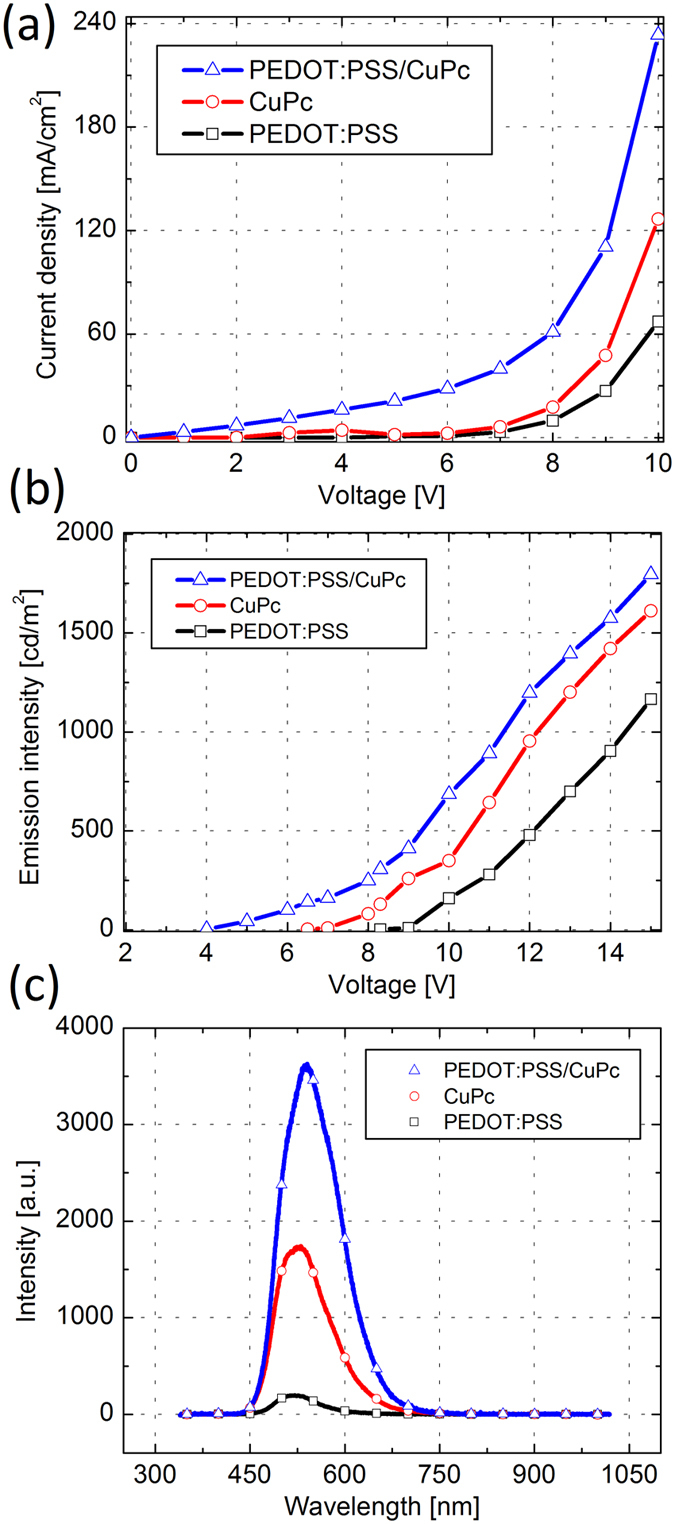
The performance characteristics of fabricated SIL and DIL based OLEDs. (**a**) Dependence of current density on bias voltage for the device with double injection layer in comparison with the reference devices with single injection layer, (**b**) emission intensity against voltage for the three devices, and (**c**) electroluminescence spectra of the devices at bias voltage of 10 V. The device with DIL presents enhanced EL spectrum.

**Figure 4:**
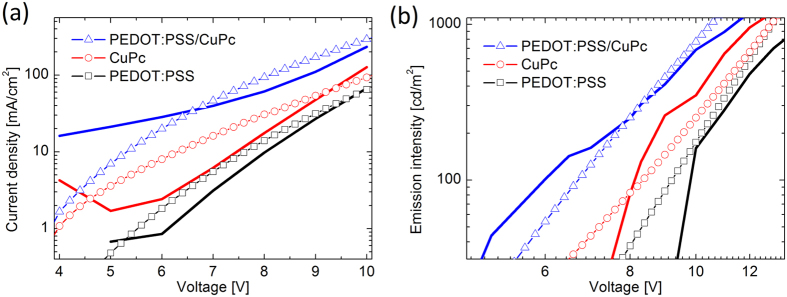
Comparison between experiment and the simulation based on computational modeling. The symbols present the results of simulation while the solid lines show experimental data. (**a**) *J-V* and (**b**) *L-V* characteristics of the three devices in logarithmic scale. The device with DIL shows improved performance.

**Figure 5:**
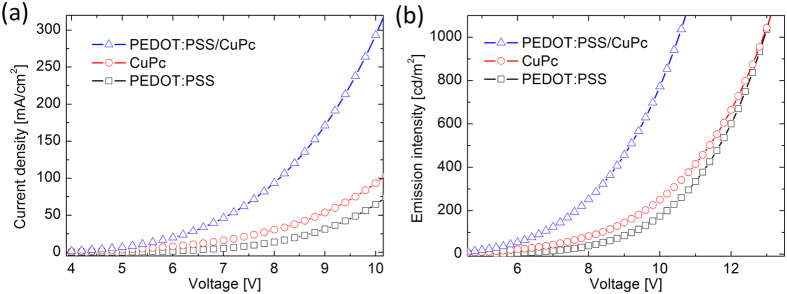
Numerical simulation of (**a**) *J-V* and (**b**) *L-V* characteristics of the device with DIL in comparison with the SIL reference devices.

**Figure 6:**
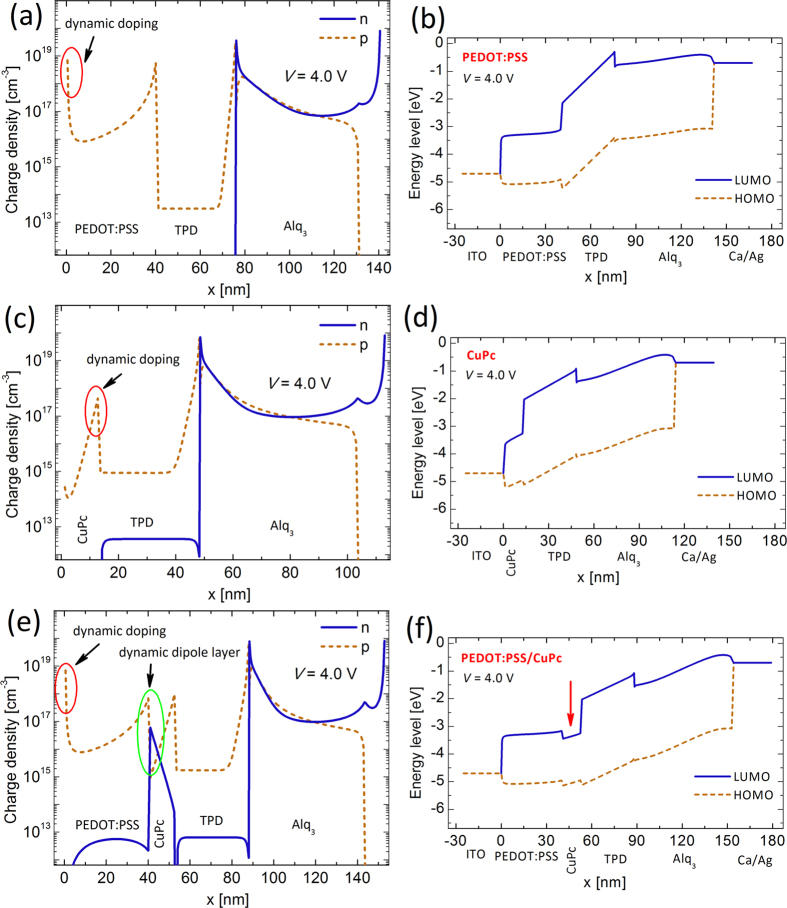
Carrier density distributions at 4.0 V for (**a**) PEDOT:PSS device (SIL), (**c**) CuPc device (SIL), and (**e**) PEDOT:PSS-CuPc device (DIL). (**b**,**d**,**f**) Energy diagrams (HOMO and LUMO levels) of the devices during operation at bias voltage of 4.0 V. Dynamic formation of charge accumulations in DIL. (**f**) The formation of quantum well in the energy diagram.

**Figure 7:**
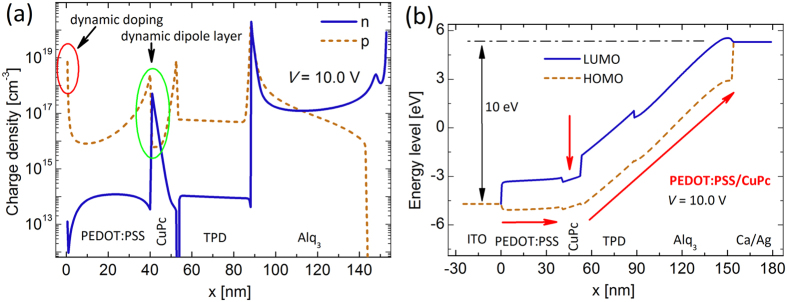
Carrier density distribution and energy level diagram for the DIL at bias voltage of 10 V. (**a**) The DDL at organic/organic interface and the associated electric field have been amplified. (**b**) The flat energy levels of PEDOT:PSS along with the high carrier density prove PEDOT:PSS acts effectively as a synthetic metal electrode with high work function in device structure.

Despite a significant increase in the carrier profiles at the interface of PEDOT:PSS-CuPc as depicted in Fig. 6e, which is in straight consistency with boosted values of current densities (Fig. 3a), the overlapping of carrier densities profiles can cause nonradiative recombination at an unfavorable location out of emission material here in Alq_3_ (Figs 6e and 7a). This can reasonably explain their lesser corresponding light intensity enhancement. The latter can be resolved by inserting an ultrathin layer of plasmonic materials; namely Ag, to sufficiently separate the carriers profiles^74^.

### Optimization strategies for OLEDs fabrication procedure

**Figure 8:**
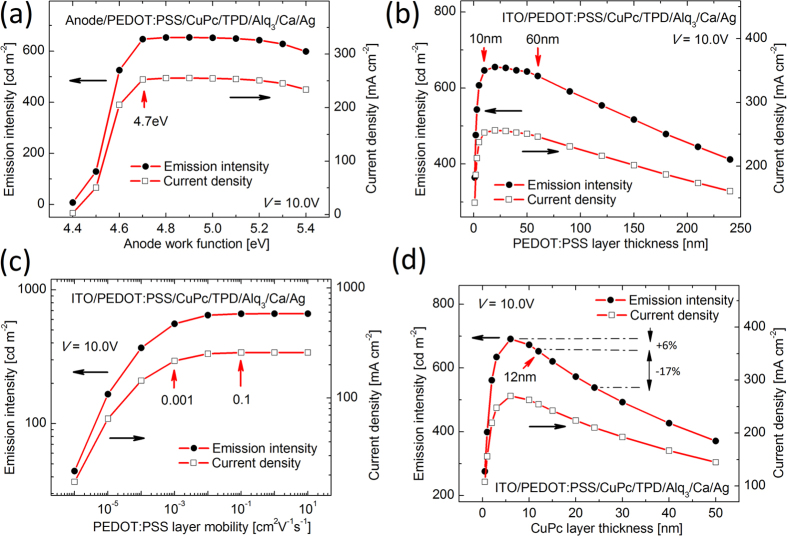
DIL device, the dependence of device performance on different parameters; namely, (**a**) the anode electrode work function, (**b**) the thickness of the first layer in double injection layer, (**c**) the mobility, and (**d**) the thickness of the second layer in double injection layer. The sensitivity and dependence of the device performance on the parameters are weakened for the DIL based OLEDs and provides latitude for device fabrication.

Heterointerface can provide several advantages and removes limitations and sensitivities for device fabrication as explained in the following. In particular, intensive device simulations were exploited in order to examine the dependency of the device performance on different parameters; namely, more than 100 devices have been simulated, which the results of 63 devices are presented here (Supporting information). Figure 8a–d demonstrate the results of simulation for the dependence of device performance on different parameters of the injection layer. The basic parameters which govern the carrier injection are the energy barriers, the mobility, and the thicknesses of the layers. As can be seen, the dependency on different parameters is weakened and therefore provides benefits for device fabrication.

Figure 8a presents the dependence of the current density and emission intensity on the work function of the anode electrode. Generally, ITO as anode needs to be treated by oxygen plasma in order to improve its work-function and reduce the energy barrier. However, all anode electrode even with work-function as low as 4.7 eV can provide sufficient current density flow into the device based on Fig. 8a. Therefore, plasma treatment step can be safely skipped from the device fabrication procedure.

**Figure 9:**
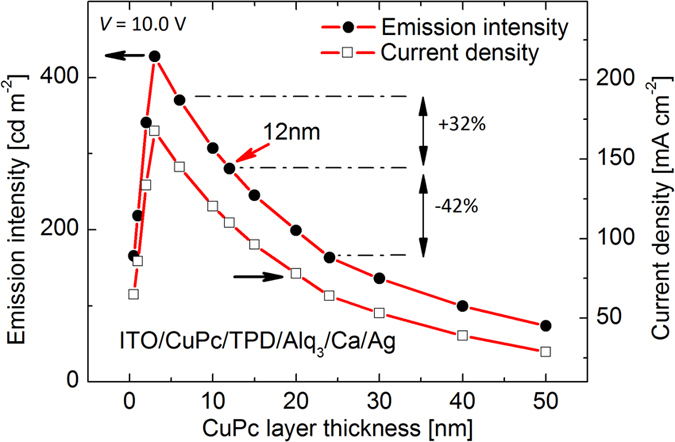
Strong dependence of device performance on the thickness of the injection layer for the device with CuPc alone as the single injection layer.

Figure 8b and d depict the dependence of device performance on the thickness of PEDOT:PSS and CuPc layers. The thicknesses of the layers determine the amount of the material consumed and therefore the device market price. Figure 8b depicts all the thicknesses between 10 nm and 60 nm for PEDOT:PSS lead to identical device characteristics. In similar manner, Fig. 8d shows assuming halved and doubled CuPc layer thicknesses results in a negligible variation in the device performance. In contrast, as seen in Fig. 9, assuming halved and doubled CuPc layer thickness in case of the device with single injection layer, the variation in device performance is considerable i.e. +32% and −42%, respectively. Consequently, the minimum adequate thicknesses can be considered in order to optimize the device fabrication process.

Finally, Fig. 8c portrays the relation between the device performance and the carrier mobility. Particularly, it is possible to enhance carrier mobility in PEDOT:PSS layer by aid of acid treatment, solvent additive, and vapor-assisted deposition. Nonetheless, the device shows reasonable performance for typical PEDOT:PSS and without any conductivity treatment, and consequently, any additional step for employing mobility treatment is unnecessary.

Unlike general experimental methods for injection enhancement^72^,^73^,^75^, we showed how simulation can systematically provide an economic and efficient strategy for general designing and optimizing of efficient DIL. It is worthwhile to note that heterointerface engineering by employing dynamic phenomena can also be exploited in other aspects such as electron injection at the cathode contact, charge generation layer in stacked structure, and in other organic devices such as organic solar cells, organic sensors, and organic filed-effect transistors.

## Conclusion

In this report, we presented an integrated computational modeling and experimental method to analyze the significant role of employing double injection layer, forming a heterointerface inejction layer, in enhancement of the carrier injection in OLEDs which is more convenient in comparison to material chemistry *i.e.* n-type/p-type molecular doping followed by OLEDs design by implementing alternate fabrication scenarios.

The results of computational modeling of fabricated devices revealed that:

- The formation of a single and double nanosheet charge accumulations in different parts of the device structure were responsible for the device performance enhancement. In particular, engineering the formation of nanosheet charge accumulations could be considered as dynamic functionalizing of the device structure. The charge nanosheet at the metal/organic interface treats the quasi E_F_ of the organic material *i.e.* DPD of the injection layer.
- The organic/organic DDL which forms a HIIL established an electric field that accelerated the carriers’ ejection and their transference to the successive layer.

Computational modeling results showed that heterointerface for carrier injection provides alternate scenarios for fabrication procedures, namely,

- no prerequisite for plasma treatment due to compatibility of the HIIL with high work-function anode electrodes (~4.7 eV–5.3 eV).
- Negligible sensitivity of device performance on the thicknesses of the materials in the injection layer *i.e.* choosing organic layer thicknesses between 10 nm to 60 nm for the first layer and between 6 nm to 24 nm for the second layer.
- Compatibility with conventional materials used for conventional SIL *i.e.* no requirement for mobility enriching owing to the compatibility of the HIIL with materials possessing moderate mobility between 0.001 cm^2^V^−1^s^−1^ and 0.1 cm^2^V^−1^s^−1^.

The proposed concept of double injection layer and dynamic formation of nanosheet charge accumulations in organic/organic heterointerface might facilitate inexpensive fabrication of OLEDs for lighting applications.

## Additional Information

**How to cite this article**: Fathollahi, M.-R. *et al*. Organic/Organic Heterointerface Engineering to Boost Carrier Injection in OLEDs. *Sci. Rep.* **7**, 42787; doi: 10.1038/srep42787 (2017).

**Publisher's note:** Springer Nature remains neutral with regard to jurisdictional claims in published maps and institutional affiliations.

## Supplementary Material

Supplementary Information

## Figures and Tables

**Table 1:** The values of main parameters employed in the model; energy levels and mobility.

Material	E^LUMO^ [ev]	E^HOMO^ [eV]	μ^n^ [cm^2^/Vs]	μ_0_^n^ [cm^2^/Vs]	F_0_^n^ [V/cm]	μ^p^ [cm^2^/Vs]	μ_0_^p^ [cm^2^/Vs]	F_0_^p^ [V/cm]
Alq_3_^47^,^51^,^61^	3.0	5.7	—	1 × 10^−8^	17500	—	1 × 10^−9^	47000
TPD^47^,^51^,^61^,^76^	2.4	5.5	—	4 × 10^−6^	360000	—	1 × 10^−5^	360000
CuPc^47^,^51^,^61^,^77^	3.6	5.3	1 × 10^−4^	—	—	1 × 10^−3^	—	—
PEDOT:PSS^78^,^79^	3.3	5.1	1 × 10^−4^	—	—	1 × 10^−2^	—	—
