# Supplementary material for: Organic/Organic Heterointerface Engineering to Boost Carrier Injection in OLEDs
Source: Sci Rep. 2017 Feb 20;7:42787. doi: 10.1038/srep42787 (PMC5316975; doi:10.1038/srep42787)
Supplement: Supplementary Information [file srep42787-s1.docx]

Supporting Information for:

**Organic/Organic Heterointerface Engineering to Boost Carrier Injection in OLEDs**

M.-R. Fathollahi^1^, M. Ameri^2^, E. Mohajerani^1^*, E. Mehrparvar^1^, and M.-R. Babaie^1^

^1^Laser and Plasma Research Institute, Shahid Beheshti University, G.C., Tehran 1983963113, Iran

^2^ Department of Physics, Bu-Ali Sina University, P.O. Box 65174, Hamedan, Iran

*Corresponding author: Ezeddin Mohajerani, E-mail: [e-mohajerani@sbu.ac.ir](mailto:e-mohajerani@sbu.ac.ir) , Telephone: +98 9121990359, Fax number: +98 2122431775

**Note:** The MOLED code was rewritten by the authors in C++ to visualize the outputs. Also, the injection equation in the model was modified by Bradeen tunneling theory as reported earlier[^1^](#_ENREF_1).

**Organic/Inorganic heterointerface:**

Heterointerface engineering to boost carrier injection is not restricted to organic/organic interfaces. In contrary, charge injection can be improved by any heterointerface with adequate energy levels and mobility. It is beneficial to investigate the idea of heterointerface engineering to enhance carrier injection with different material combination. For instance, it is worthwhile to investigate OLEDs which employed MoO_3_ hole injection layer. Therefore, we examined two more devices based on MoO_3_. Following the structures investigated in the paper, the first device uses a single layer of MoO_3_ between ITO and TPD, while the other one exploited a heterointerface based on PEDOT/MoO_3_ combination. In other words, two more devices have been investigated similar to the ones presented in the paper in which CuPc was replaced by MoO_3_. Figure S1 compares the current-density versus voltage and the emitted light versus voltage characteristics of all the five devices; three of them employed single injection layer and the other two used heterointerface injection layer.

According to the figure, it could be observed the device employed single MoO_3_ injection layer shows higher performance in comparison with the two devices which exploited single CuPc (hallow circle) or single PEDOT:PSS (hallow square) as hole injection layer. However, it could be noticed one more time that the device employed heterointerface injection layer *i.e.* PEDOT:PSS/MoO_3_ (solid triangle) shows superior performance than that of the one employed single MoO_3_ hole injection layer (solid circle) in accordance with the presented results in the paper.

There are a number of studies which investigated devices including MoO_3_ in adjacent to PEDOT:PSS or the combination of the two. Despite that structure of the devices studied in the literature are not exactly the same as the ones investigated here; however, the similarity between the results is of much interest. The appendix describes some examples briefly.


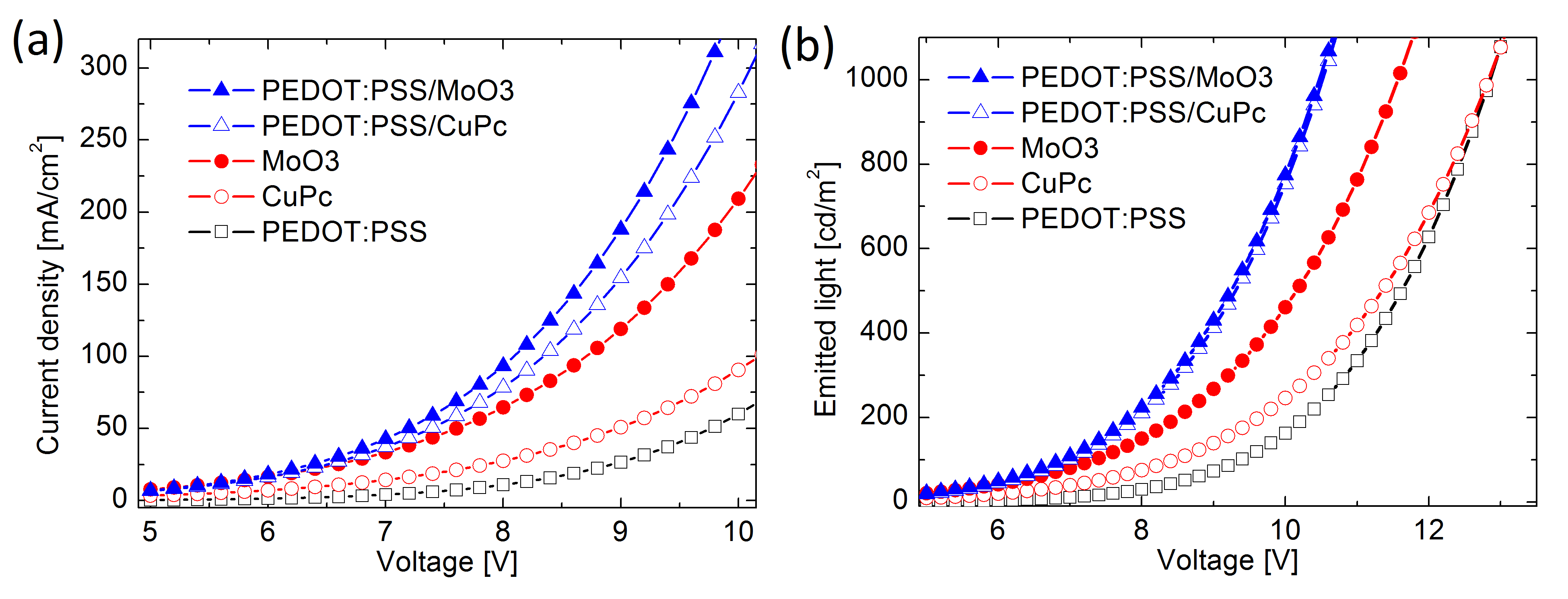


Figure S1 Comparison between (a) *J-V* and (b) *L-V* characteristics of devices employed different SIL; namely, PEDOT:PSS, CuPc, and MoO_3_ and different HIIL; namely, organic/organic heterointerface of PEDOT:PSS/CuPc and organic/inorganic heterointerface of PEDOT:PSS/MoO_3_. The devices with double injection layer present superior characteristics in comparison with the control SIL devices.

As mentioned previously, the underlying physics at microscopic level provides a more reasonable description for the device performance improvement. Figures S2.a and S3.a illustrate the charge carrier distributions within the organic layers for the both devices, the one employed MoO_3_ alone and the one employed PEDOT/MoO_3_ heterointerface as hole injection layer, respectively. In similar manner to that proposed for the device made with PEDOT/CuPc heterointerface, two dynamic phenomena join forces to enhance carrier injection in device made with PEDOT/MoO_3_ heterointerface *i.e.* (a) dynamic doping of the organic material near anode electrode and (b) dynamic dipole layer at the heterointerface, here organic/inorganic, due to opposite charge accumulation across the interface. Figures S2.b and S3.b present the energy band diagram for both devices, the first one with MoO_3_ as single injection layer (SIL) and the second with PEDOT/MoO_3_ as heterointerface injection layer (HIIL), respectively. As can be seen, a deep quantum potential well is formed in the energy level diagram which leads in large number of electron accumulation within MoO_3_ layer (Figure S3.b).

Various values have been reported for the energy levels of MoO_3_. For example, Table 1 tabulates some of the documented values for energy levels of MoO_3_ adapted from reference 2 [^2^](#_ENREF_2). Despite the difference in reported values for MoO_3_; however, all of them are still large enough compared to HOMO level of the adjacent organic layers within the structure. Consequently, it could be considered the conduction band energy level of MoO_3_ is available not only for the transport of electrons between the adjacent layers but also is available to the holes (Figure S4). Therefore, it might be assumed for the simulation that both LUMO and HOMO of MoO3 layer to be, for instance, 5.5eV. Regarding carrier mobility in MoO_3_, the value is large in MoO_3_ [^3^](#_ENREF_3). Therefore, it seems reasonable to assume large value for carrier mobility in MoO_3_ in comparison with common organic materials used in OLEDs. We assumed the carrier mobility to be 1cm^2^V^-1^s^-1^ in MoO_3_ (Please see reference 3 and its supporting information particularly the mobility of MoO_3_ in bulk).


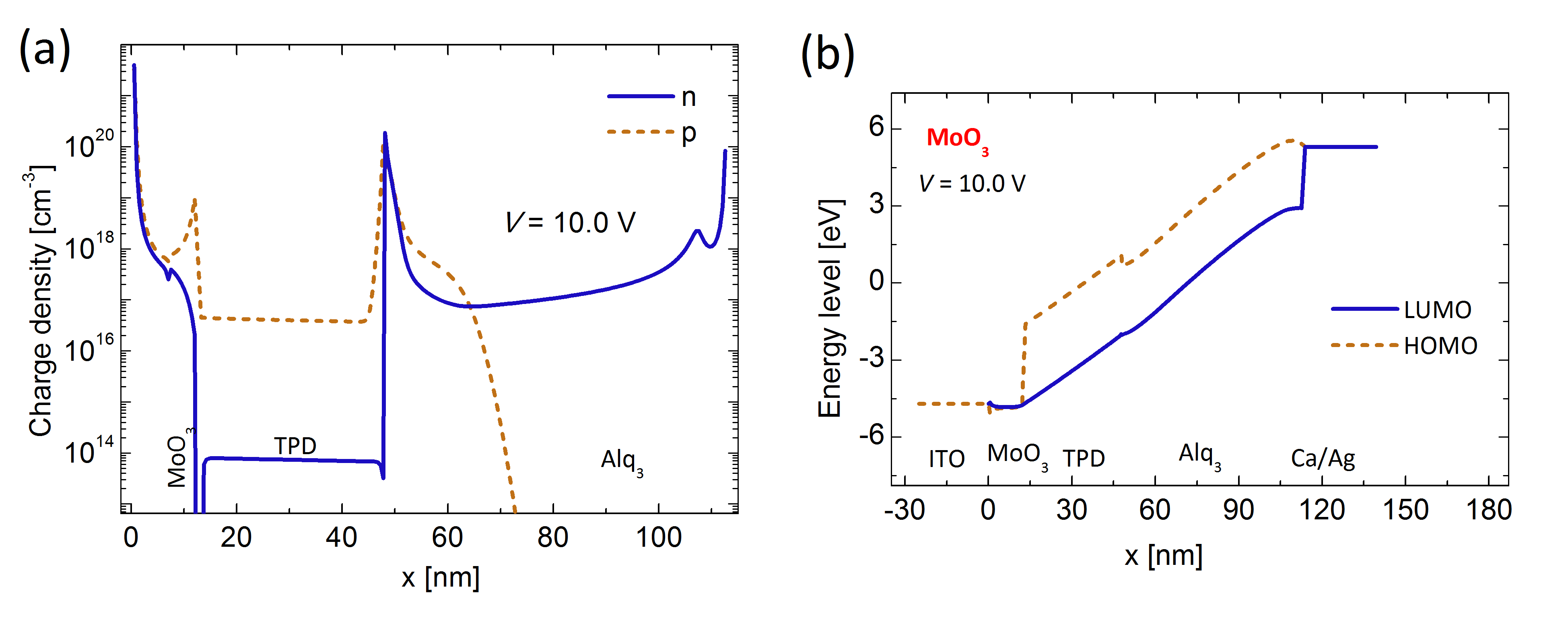


Figure S2 (a) Carrier density and (b) energy level diagram for device employed MoO_3_ as single injection layer.


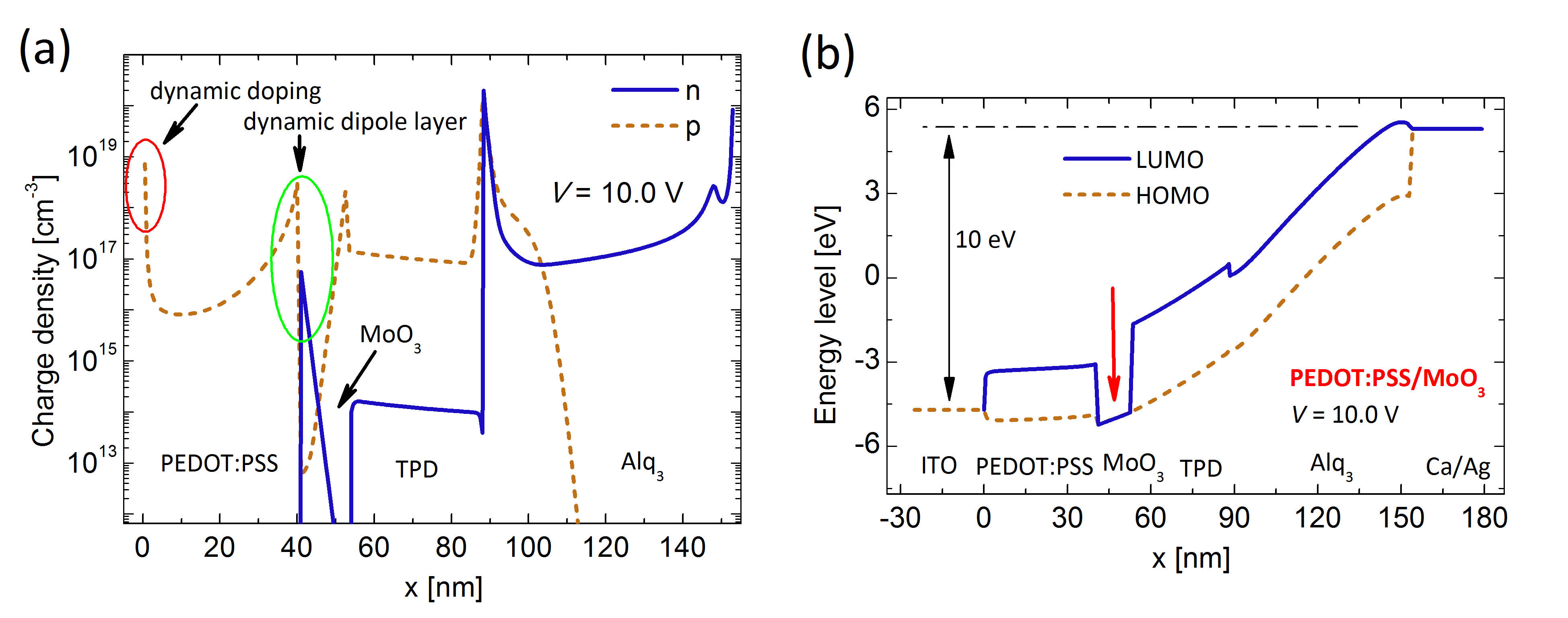


Figure S3 (a) carrier density distribution and energy level diagram for device employed PEDOT:PSS/MoO_3_ organic/inorganic heterointerface as hole injection layer, (a) dynamic formation of charge accumulation and (b) quantum well potential within energy level diagram.

Based on experimental results, please see the appendix, MoO_3_ as hole transport layer shows reasonably high performance. The simulation results strongly confirm the claim. Figure S5 presents the dependence of carrier density and emitted intensity on the electron affinity of MoO_3_ layer for a device employed MoO_3_ as single injection layer. The variation of energy level might be ascribed to the change in the oxygen stoichiometry as exposed to ambient air or to be due to the different synthesis and deposition process condition of MoO_3_ layer. It could be seen whenever the conduction band energy level of MoO_3_ is lower than that of adjacent layer, the MoO_3_ layer acts an effective hole injection layer which is the case in practical situation. Figure S6 and S7 also illustrate the dependence of device performance on other structure parameters, namely layer thickness and electrode work function, respectively. Referring to figures, the device made with MoO_3_ offers excellent performance in accordance with experimental results reported in the literature.


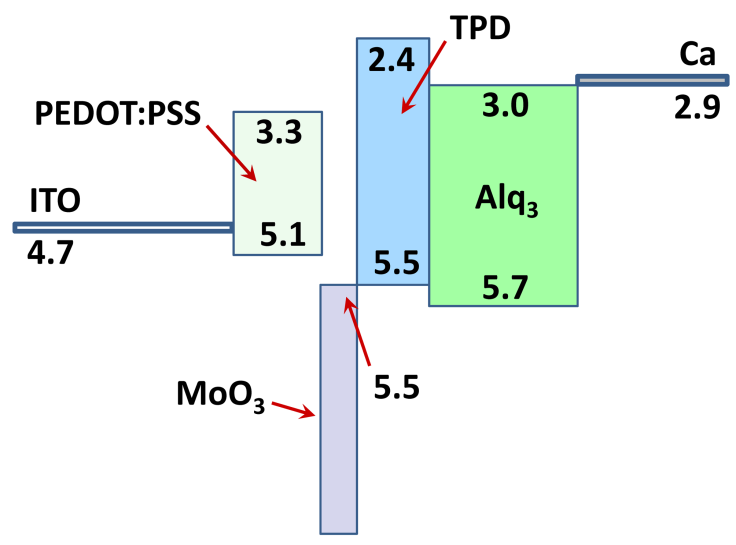


Figure S4 schematic band diagram of the device employed PEDOT:PSS/MoO3 as double injection layer *i.e.* organic/inorganic heterointerface injection layer.

Table S1. Reported values for energy levels of MoO_3_. Reproduced from Ref 2 with permission of The Royal Society of Chemistry[^2^](#_ENREF_2).

| Process | Post-treatment | W_F_ [eV] | EA [eV] | IE [eV] |
| --- | --- | --- | --- | --- |
| Evaporation | - | 6.7 | 6.9 | 9.5 |
|  | In ambient air | 5.7 (4.9) | 5.5 | 8.6 |
| Nano-particle | In ambient air | 5.4 | 4.9 | 8.3 |
|  | In ambient air, O_2_-plasma, annealing | 6.0 | 5.8 | 8.9 |
| Sol-gel | O2-plasma, annealing | 6.1 | 5.5 | 9.0 |
|  | Annealing in N_2_ | 5.4 | 4.3 | 8.0 |
|  | In ambient air | 4.9 | 4.8 | 7.9 |
|  | In ambient air, annealing in N_2_ | 5.3 |  |  |


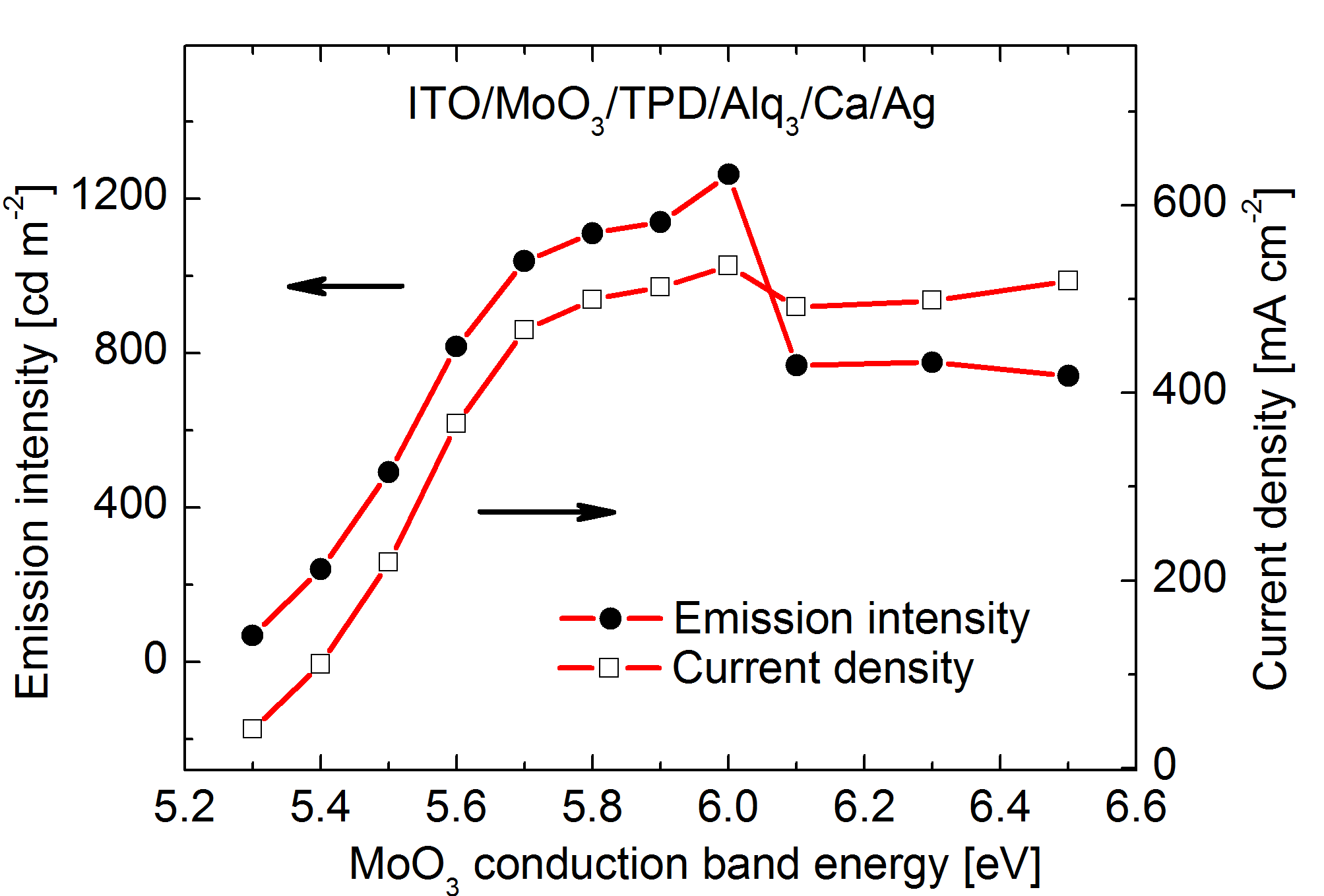


Figure S5. Current-density and emitted light against electron affinity energy of MoO_3_ layer. The device performance decreases by variation of the effective energy level of the material due to the change of the oxygen stoichiometry within the layer. Nevertheless, the layer acts as an efficient hole injector for a wide range of energy level values.


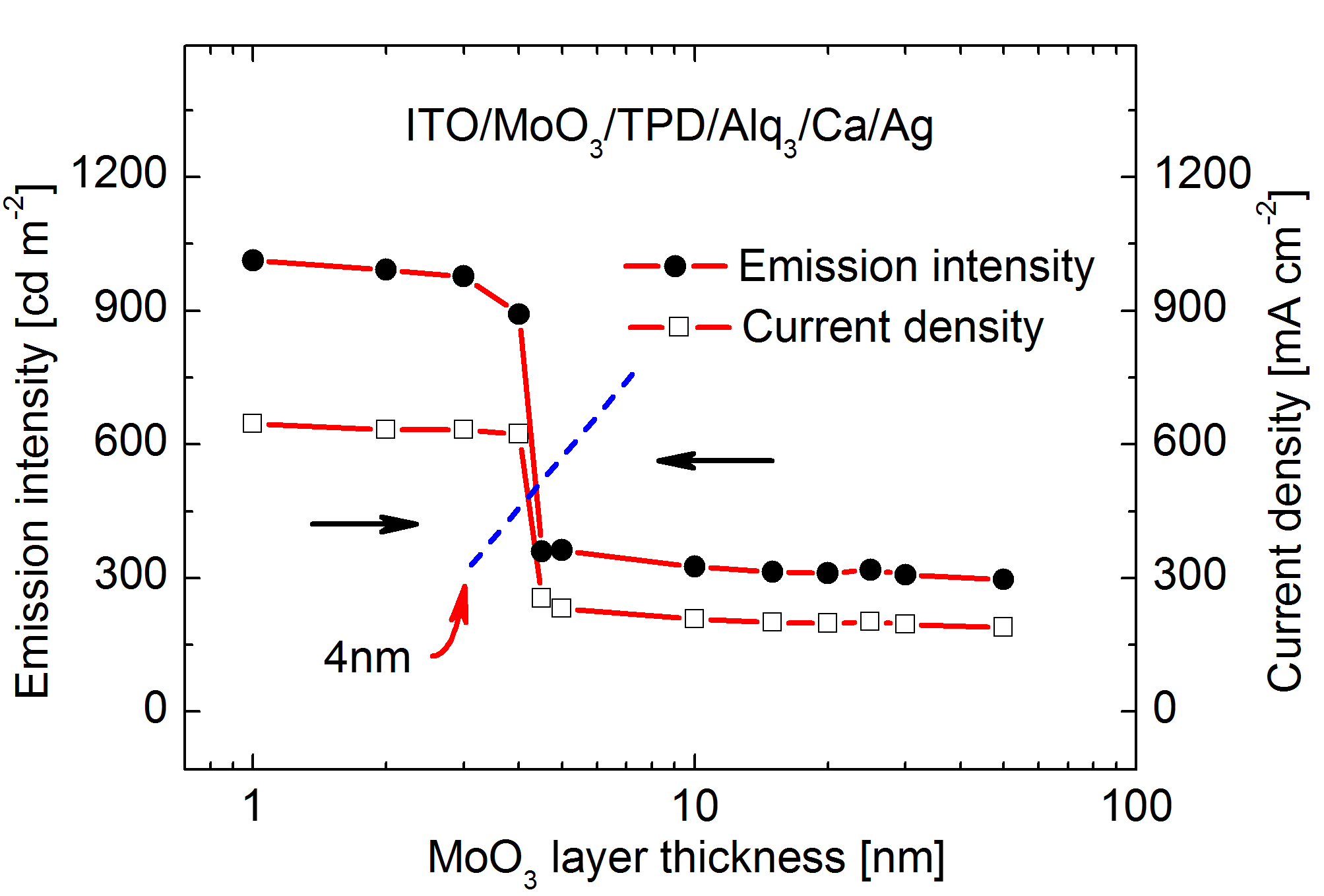


Figure S6. The dependence of current density and emitted light on the thickness of MoO_3_ layer. It is noticeable that according to simulation results a transition takes place in injection regime into tunneling injection with an increase in current density flowing through the device.


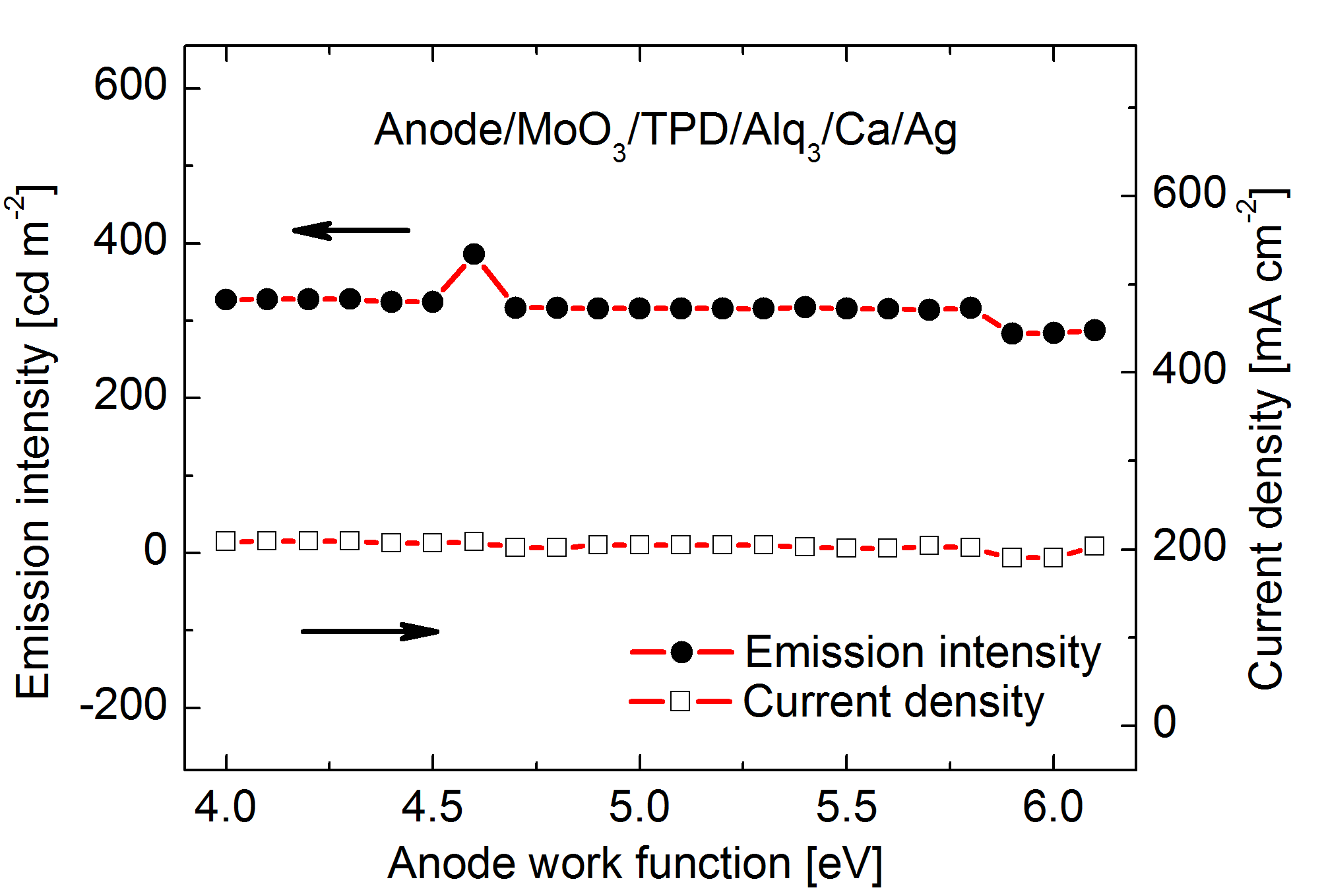


Figure S7. The dependence of current density and emitted light on anode work function for device employed MoO_3_ as single injection layer. MoO_3_ layer acts as an effective injection layer for a wide variety of electrodes with different work function.

**The physical model:**

As mentioned, a comprehensive numerical model called MOLED was employed in order to simulate the characteristic improvement in OLED devices with double injection layers[^1^](#_ENREF_1)^,^[^4-10^](#_ENREF_4) . Basically, the model developed based on the fact that charge carrier states are localized in organic materials. As depicted in Figure S8, an effective one-dimensional (1D) array of discrete nodes represents the device in the model, in which the axis is perpendicular to the anode and cathode electrodes. The charge distributions within the organic layers are presented by *n*_m_ for electrons and *p*_m_ for holes, where m=1, …, N are associated to the organic layers and m=0 and N+1 stand for the anode (the left site) and the cathode (the right site), respectively. The model solves the dynamic of the charge carriers which consists of three contributions, the injection of the carriers from the electrodes to the organic bulk, the transport of the carriers among the organic molecules, and finally the electron-hole recombination as follows:


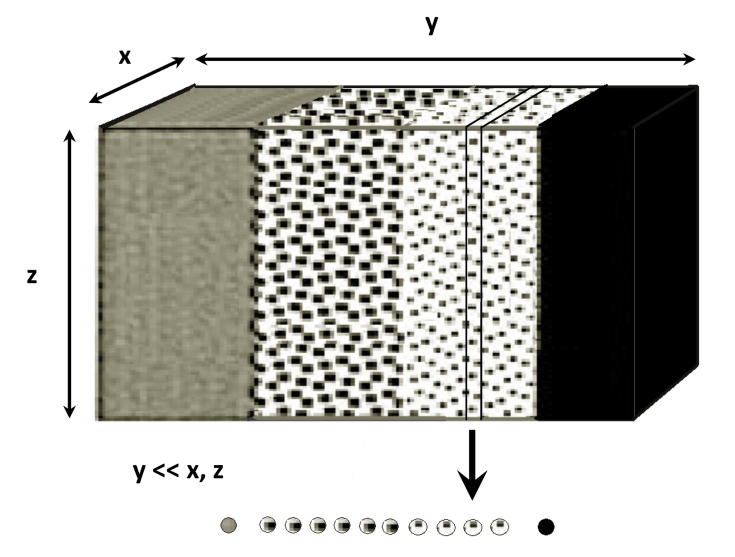


Figure S8. An effective one-dimensional (1D) array of discrete nodes represents the device in the model.

$\begin{matrix} \frac{dn_{m}}{dt}=\left(\frac{dn_{m}}{dt}\right)_{inj}+\left(\frac{dn_{m}}{dt}\right)_{hop}+\left(\frac{dn_{m}}{dt}\right)_{rec} \\ \frac{dp_{m}}{dt}=\left(\frac{dp_{m}}{dt}\right)_{inj}+\left(\frac{dp_{m}}{dt}\right)_{hop}+\left(\frac{dp_{m}}{dt}\right)_{rec} \end{matrix}$ (s1)

**Carrier transport within the organic layers:**

The carrier transition among the organic molecules with localized states can be modeled by considering the master equation *i.e.* via hopping between the nearest-neighboring sites:

$\begin{matrix} \left(\frac{dn_{m}}{dt}\right)_{hop}=\Omega_{m}\left[-n_{m}e^{(E_{m}-E_{m-1})/2k_{B}T}+n_{m-1}e^{(E_{m-1}-E_{m})/2k_{B}T}\right] \\ \text{ -}\Omega_{m+1}\left[-n_{m+1}e^{(E_{m}-E_{m+1})/2k_{B}T}+n_{m}e^{(E_{m+1}-E_{m})/2k_{B}T}\right] \end{matrix}$ (s2)

where *Ω_m_* is the hopping frequency between the sites *m* and *m*-1, *E_m_* the energy level of the site *m*, *T* the temperature, and *k_B_* the Boltzmann’s factor. The effective hopping frequency within the organic materials can be adapted from the mobility measurements:

$\Omega=\frac{1}{qa^{2}}\mu\frac{\left(Fqa/2k_{B}T\right)}{\sinh\left(Fqa/2k_{B}T\right)}k_{B}T$ (s3)

where *a* is the distance between the carrier sites (the organic molecules), *F* the external electric field, and *q* the elementary charge. Generally, charge transport and carrier mobility in organic materials are described by various models such as small polaron hopping model, Poole–Frenkel model, Gaussian disorder model, correlated Gaussian disorder model, symmetric microbalance model, asymmetric microbalance model and so on. In the code, four mobility models are formulated [^7^](#_ENREF_7)^,^[^9^](#_ENREF_9).

**Carrier recombination in the emissive layer:**

The recombination process can be simply modeled by Langevin recombination formula:

$\left(\frac{dn_{m}}{dt}\right)_{recom}=-r\times p\times n;\text{ }r=q(\mu_{n}+\mu_{p})/(Aa\varepsilon_{0}\varepsilon_{r})$ (s4)

in which *A* is the organic molecule size in perpendicular direction, *μ*_n_ and *μ*_p_ the electron and hole mobility, *ε*_0_ the vacuum permittivity, and *ε*_r_ the relative permittivity. The recombination formula can be modified to include the effect of diffusion current as well.


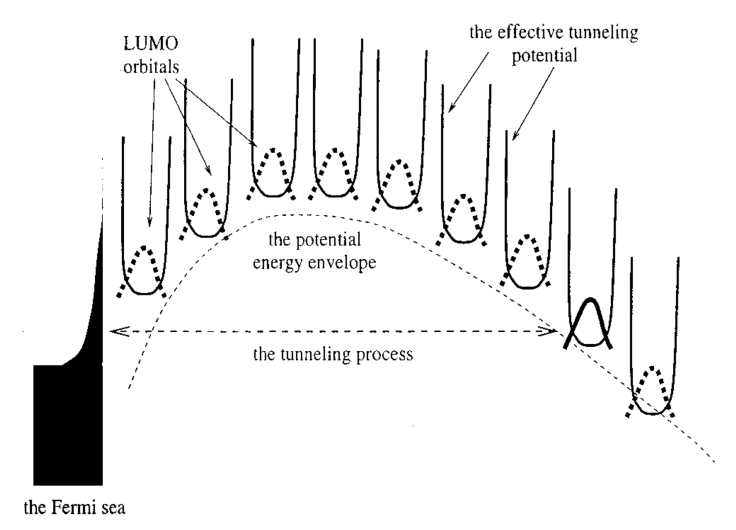


Figure S9. Carrier injection at metal-organic contact. Reprinted from Tutiš, E., Bussac, M.-N., Masenelli, B., Carrard, M., and Zuppiroli, L., Journal of Applied Physics, 89, 430-439, (2001), with the permission of AIP Publishing[^5^](#_ENREF_5).





Figure S10. Potential profiles to explain the carrier injection by employing Bardeen tunneling theory. Reprinted from Organic Electronics, 13, Fathollahi, M.-R., Boroumand, F. A., Raissi, F., and Sharifi, M.-J., Quantitative characterization of carrier injection across metal–organic interfaces using Bardeen theory, 905-913, Copyright (2012), with permission from Elsevier[^1^](#_ENREF_1).

**Carrier Injection from the Electrodes:**

Carrier injection from the electrodes can be formulated through tunnelling process. Therefore, the carrier transmission rate can be expressed in terms of a tunnelling integral between the electrode and the adjacent organic node and also by considering a decay factor to account for the intersite tunnelling (Figure S9) [^7^](#_ENREF_7)^,^[^9^](#_ENREF_9)^,^[^11^](#_ENREF_11). Equivalently, the carrier transition between the electrode and the organic molecules can be modeled by Bardeen tunneling theory as depicted in Figure S10 and in particular the hopping frequency for the transition rate can be expressed as follows[^1^](#_ENREF_1)^,^[^12^](#_ENREF_12):

 (s5)

where *n_n_* is the charge density, *f* the Fermi-Dirac function, *ν_n_* the hopping rate, *M* the matrix element, *ħ* the reduced Planck’s constant, and *ρ* the density of states per unit length. The *α* shows carrier wave’s amplitude attenuation caused by passing through the narrow barriers in the organic materials and the term *exp(-2d(x_n_))* includes the effect of slow variation in potential profile. The term *ν_0_* has the unit of *s^-1^* and express the hopping rate between metal and organic molecules, in which *L_well_* is the carrier delocalization length in the organic semiconductor, *m_e_* the electron mass, *α’* the wave function attenuation at the contact caused by the barrier, and finally *K_m_*, *K_n_*, *κ*, and *κ’* are the carrier wave vectors in different regions of the metal-organic contact.

For the electrodes, the tunneling to all the molecular sites and also the connection to the external circuit should be considered:

$\left(\frac{dn_{0}}{dt}\right)=-\sum_{m=1}^{N} \nu_{_{m}}^{n}\left\{f-n_{m}\right\}+\sum_{m=1}^{N} \nu_{_{m}}^{p}\left\{f-p_{m}\right\}+\left(\frac{dn_{0}}{dt}\right)_{bat}$ (s6)

The term (d*n*_0_/dt)_bat_ *i.e.* the current in the external circuit *J*_bat_ *A* = - q (dn_0_/dt) = q (dn_N+1_/dt) is calculated in a way that the energy levels at the electrodes follows the applied bias voltage:

 (s7)

**Molecular energy levels during device operation:**

The carrier energy levels at site *m* are influenced by the charge accumulation within the organic layers and the charges at the electrodes[^7^](#_ENREF_7):

 (s8)

where *x*_m_ is the position of the site *m*, *E*_0m_ the bare molecular energy level, *x*_RI_ = *x*_N+1_ + (*x*_N+1-_*x*_N_), *r* the discreteness radius parameter for the first and the last organic monolayers, and *E*_IF_ is the image force potential which can be written as:

$E_{IF}\left(x_{m}\right)=-\frac{q^{2}}{16\pi\varepsilon_{0}\varepsilon_{r}x_{m}}-\frac{q^{2}}{16\pi\varepsilon_{0}\varepsilon_{r}\left(L-x_{m}\right)}$ (s9)

**Implementation of the model:**

The described numerical model was implemented in C++ developer environment based on the original Fortran code (Figure S11). As a result, a computer program is released, **oled.exe**, and is employed to study the device with double injection layer and practically to investigate the improvement in the device characteristics (Figure S12). To start the simulation, the developed computer program needs an input file, in which the device structure, material parameters, device parameters, and also simulation parameters are described (Figure S12).

**
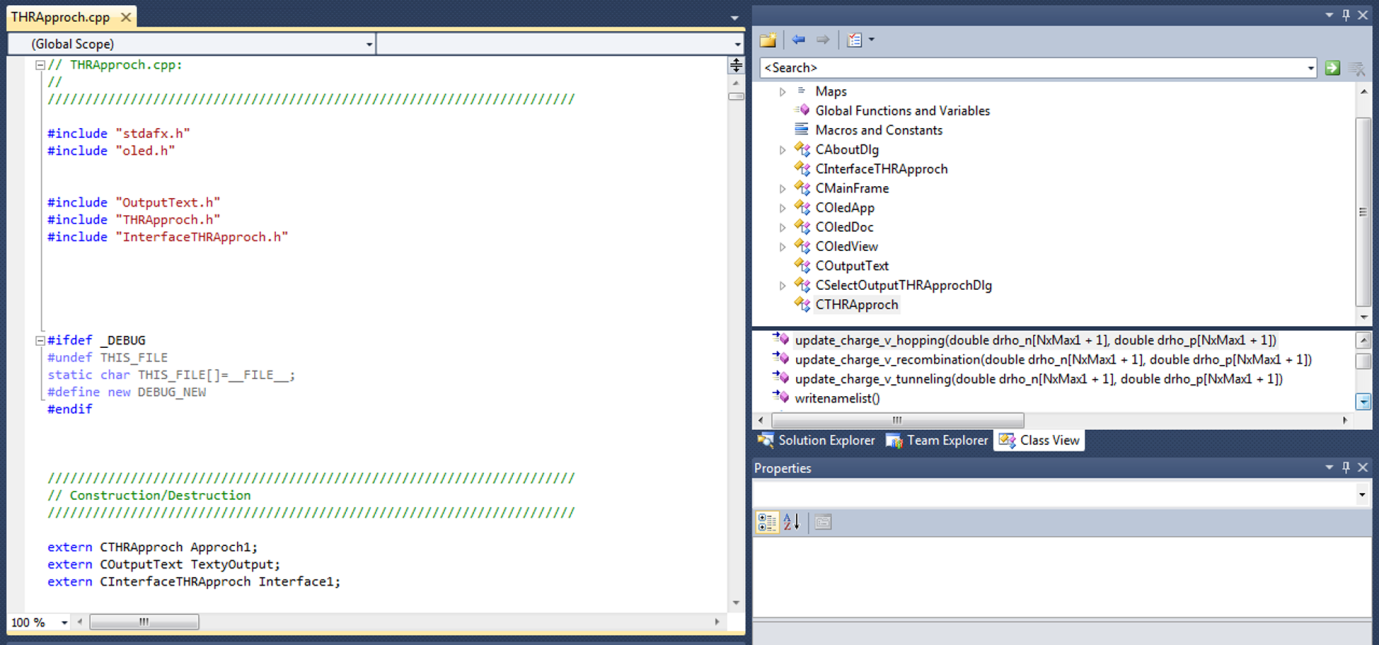
**

Figure S11. Implementation of the numerical model in C++ developer environment.

**
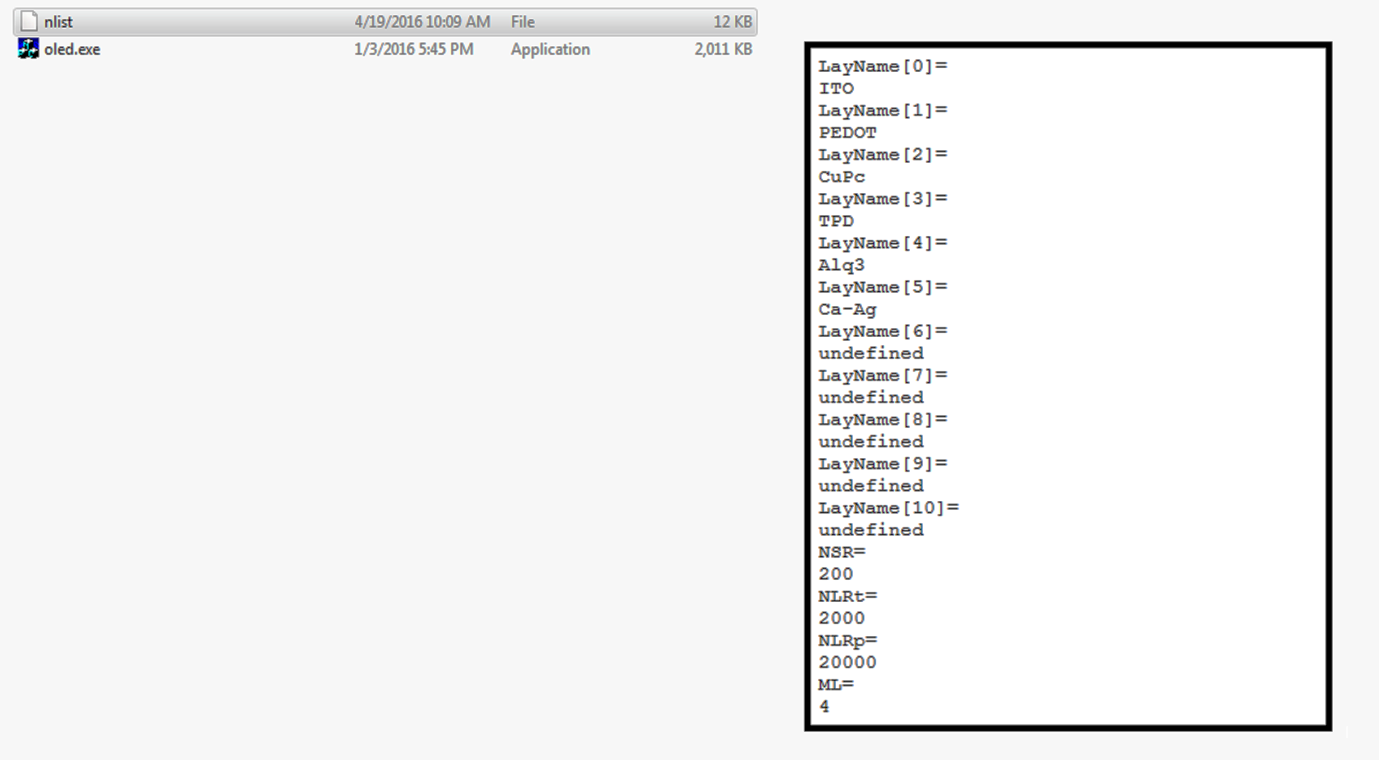
**

Figure S12. The computer program, oled.exe, and input file called nlist.

**
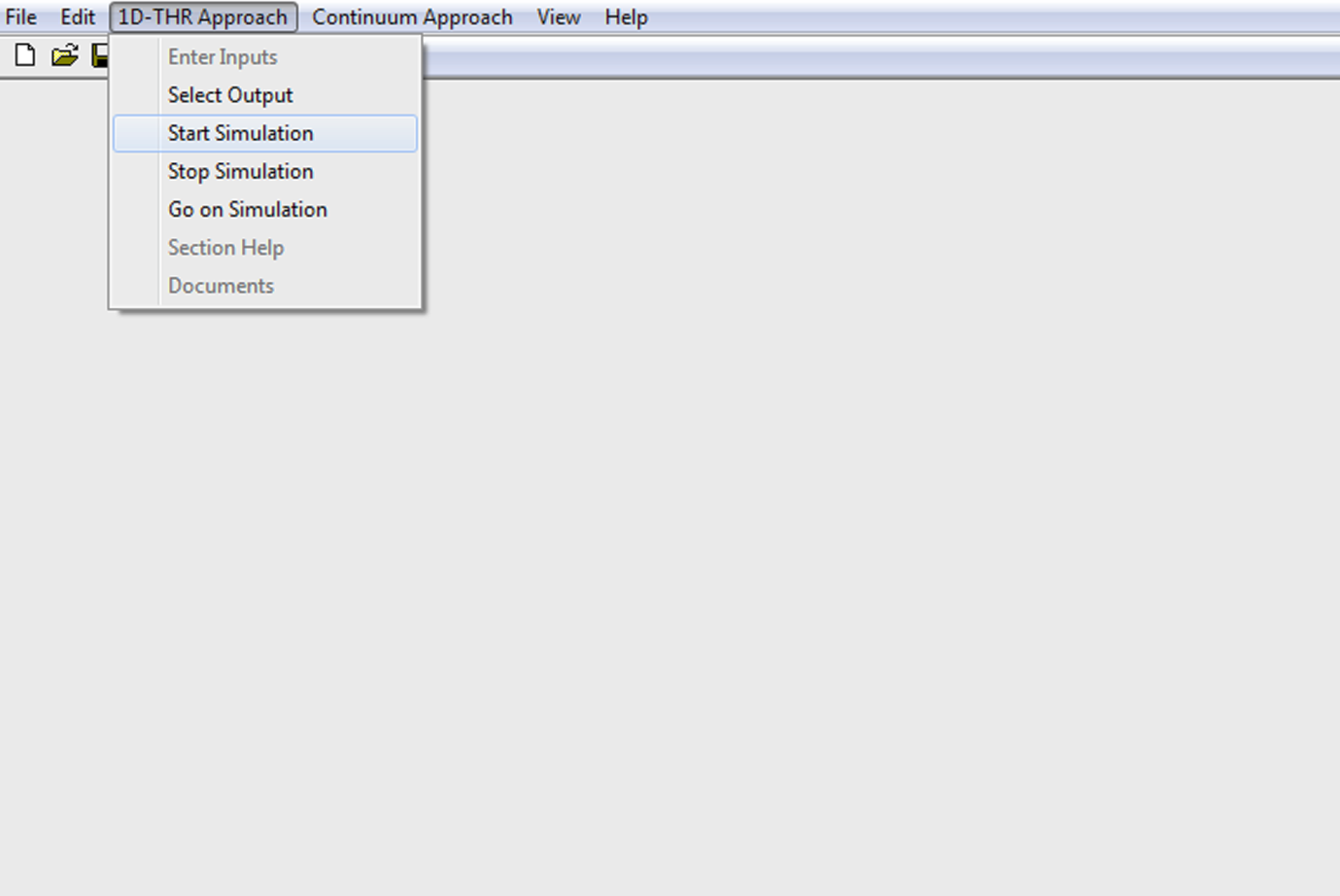
**

Figure S13. The simulation can be started by selecting the “start simulation” entry from the “1D-THR approach” menu.

After defining the device structure and determining the material parameters and also simulation parameters in the input file, numerical simulation can be started by double-clicking the program icon and selecting the “start simulation” entry from the “THR Approach” menu as shown in Figure S13.

The computer program generates several output files in analogy with in the original Fortran code. Basically, the computer simulation provides two types of output data, (a) macroscopic device characteristics, and (b) microscopic quantity profiles. Mainly, the distributions of the physical quantities at microscopic scale within the organic layers are recorded in separated output files as the time is advanced *i.e.* the condition of each organic site. Lastly, the macroscopic characteristics of the simulated device are recorded in a separated output file (Figure S14).


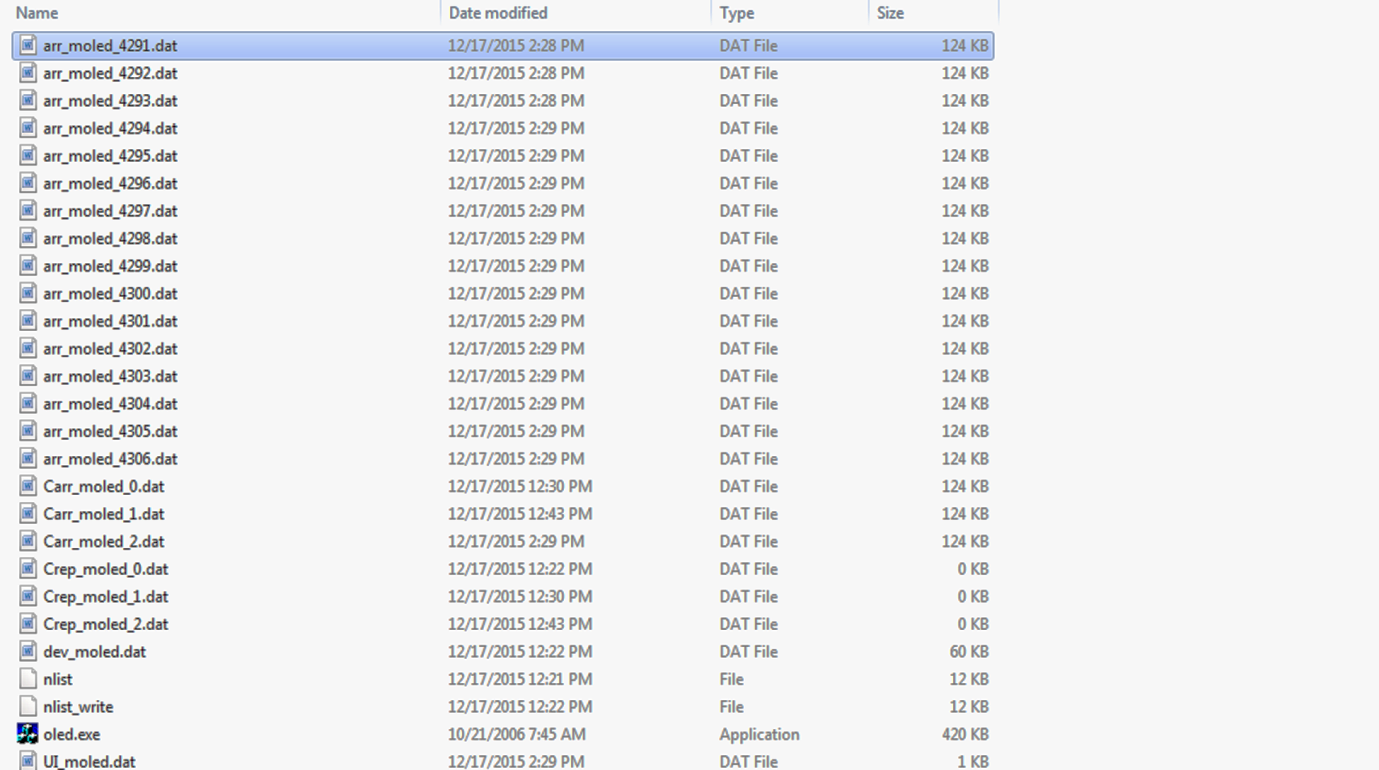


Figure S14. Several output files are generated during the device simulation.

Generally, the device simulation is performed for a set of given bias voltages. For each bias voltage, the numerical simulation is followed through successive iteration in time. During each iteration, the values of physical quantities are calculated for each organic node and for the electrodes as well. Namely, the charge densities for the next step are calculated by considering the hopping term, the injection term, and the electron-hole recombination term. Then, the energy levels at each organic site are calculated by considering the space charge within the organic layers. Afterwards, different components of the current density are calculated at each node. The iteration is repeated until the stationary state condition is reached for the given bias voltage.

As mentioned, several output files are generated during the device simulation, in which the values of the physical quantities for each organic node are recorded. The output files for the microscopic quantities are written on hard disk at given time steps, which can be used to trace the device transient behavior. Also, the microscopic quantities are saved in separate files for each bias voltage when the steady state condition is reached. Figure 15.a-15.e present the details of an output file, which contains the values for the microscopic quantities for each organic node.

The microscopic physical quantities which are recorded in the output file are as follows: electron density, hole density, electron mobility, hole mobility, electron molecular orbital energy level, hole molecular orbital energy level, local electric filed, different components of the current density, and the current contribution to the electron-hole recombination.


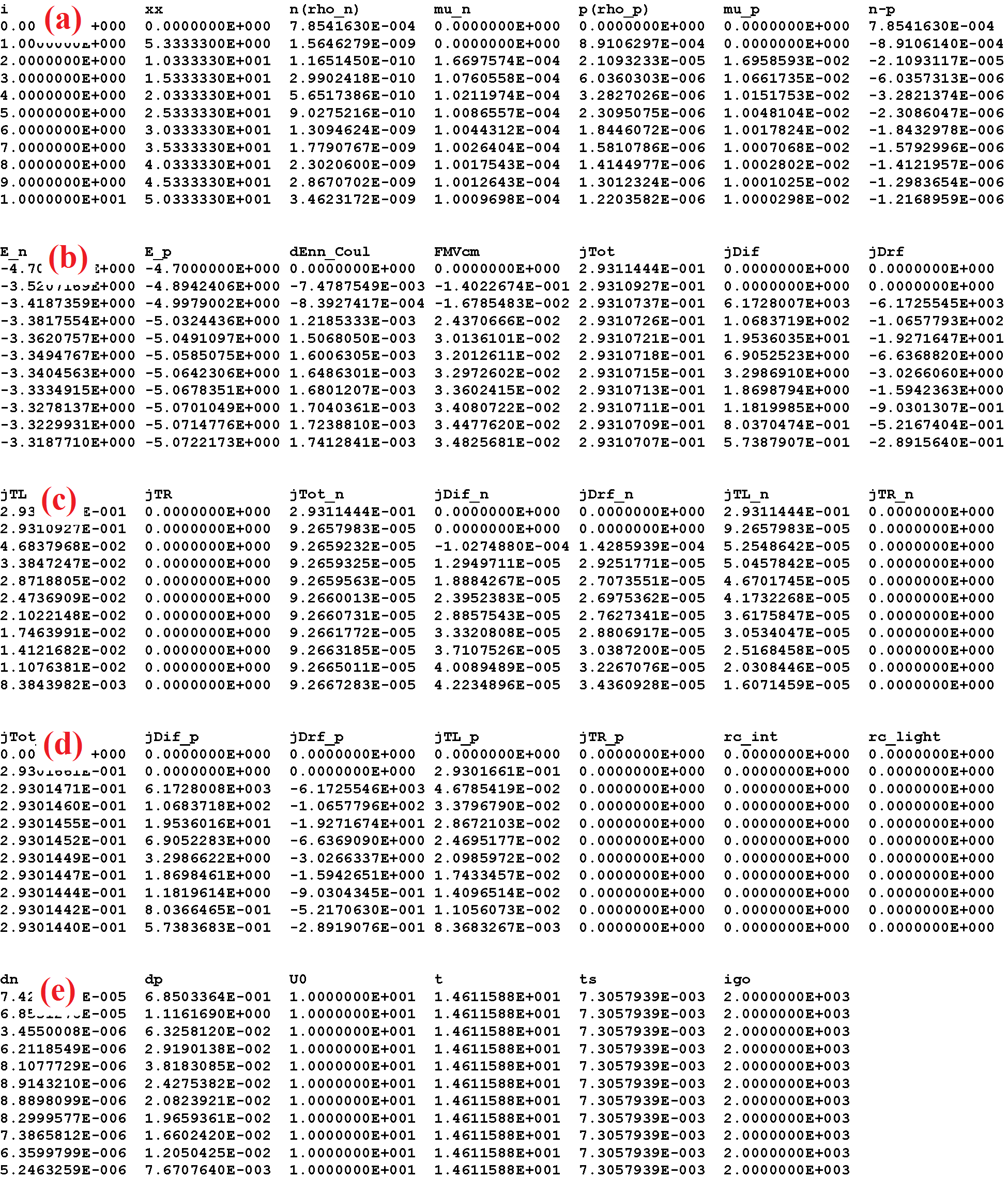


Figure S15. Carr_moled_25_record for V=10.0.txt output file. The computer program has generated this output file when the stationary state condition is reached for the bias voltage of 10.0V. The file contains the values of the microscopic quantities for each organic node.

Finally, the device characteristics are saved in a final output file; namely, the applied bias voltage, the total current density, the total emitted light, and the device efficiencies. For instance, Figure 16.a-16.c present the generated output files, which contain the macroscopic device characteristics for the three


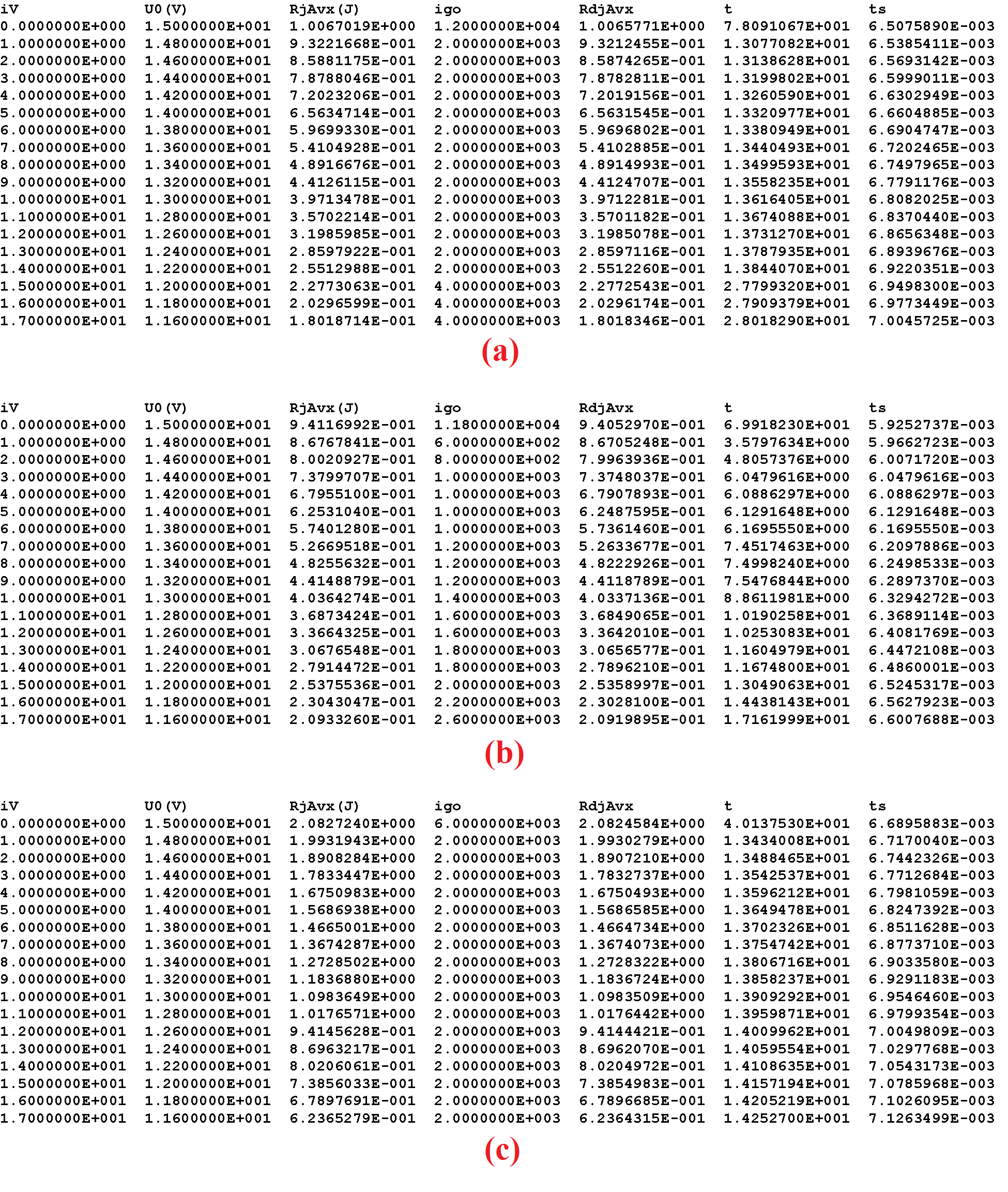
Figure S16. UI_moled.txt output files for the three devices. The computer program generates these output files at the end of the device simulation. The file contain the device macroscopic characteristics i.e. the applied bias voltage, the total current density, the total emitted light, and the device efficiencies, (a) PEDOT alone, (b) CuPc alone, and (c) double injection layer device.

devices; namely, PEDOT:PSS-alone, CuPc-alone, and the device with double injection layer. When, the simulation is finished, the output files can be imported into plotting packages in order to illustrate the simulation results. For example, Figure 4 in the paper and Figure S1 in this supporting information have been drawn as described.


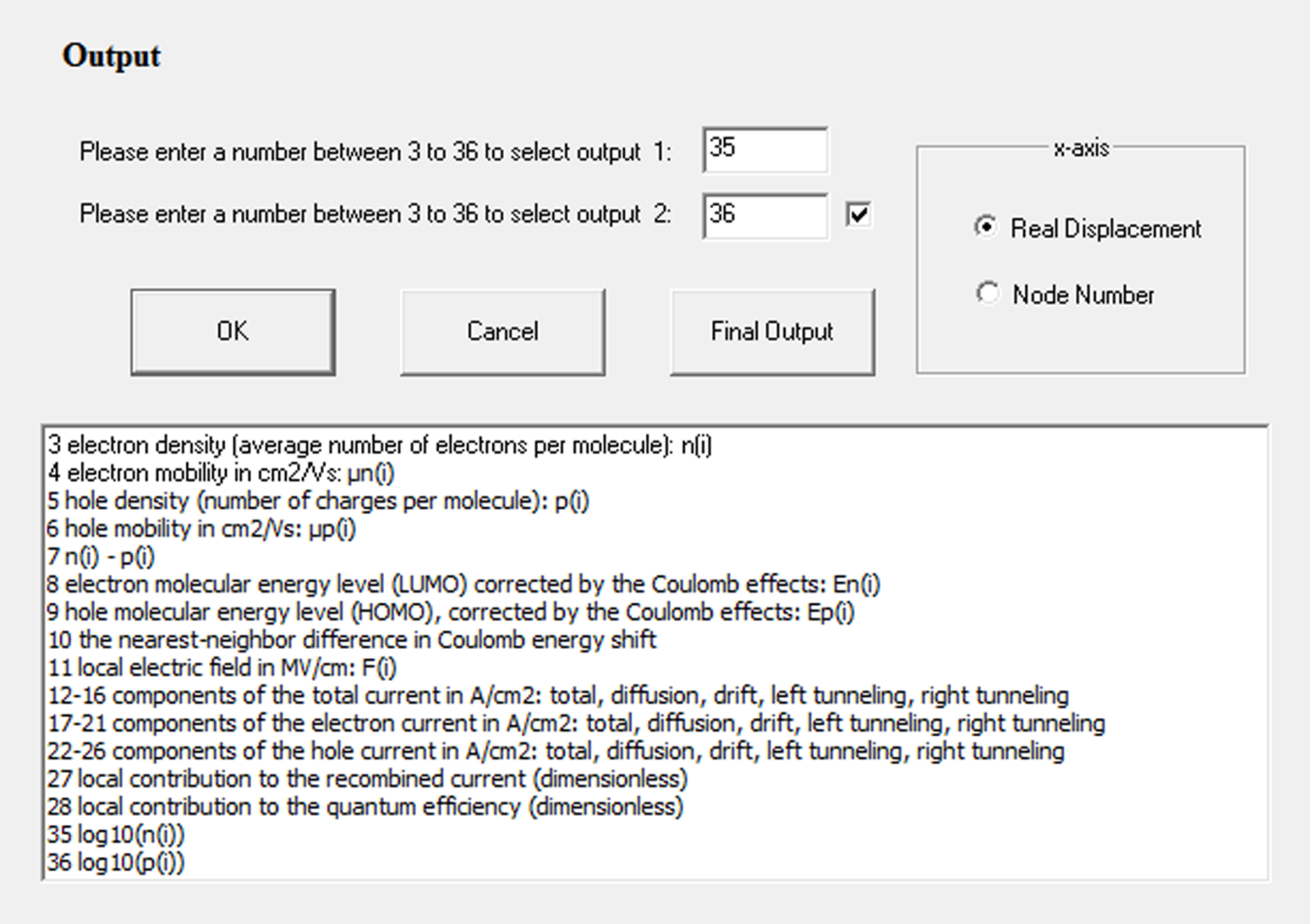


Figure S17. Selecting the physical quantities to be traced and visualized during the simulation.

Regarding the underlying physics of the device operation, the implementation of the numerical model by Visual C provides beneficial advantages. The developed code can visualize dynamically the time evolution of the physical quantities during the device simulation. Particularly, various microscopic quantities can be chosen to be traced during the simulation (Figure S17).

During the simulation, at any time, the computer program can be stopped in order to take snapshots from the physical quantity profiles along the axis perpendicular to the electrodes. Then, the simulation can be continued. When the bias voltage is applied across the device, the electron from the cathode and the holes from the anode are injected into the bulk of the device. Afterwards, the negatively charge carriers and positively charge carriers *i.e.* the polarons are transported towards the emissive layer under the application of the external electric field. Therefore, the charge carrier profiles are built-up from the injecting electrode towards the opposite electrode. Nonetheless, the variation in the electron and hole molecular energy levels along the organic layers introduce potential barriers and potential wells for the transport of the charge carriers. The set of the following figures (Figure S18) present step-by-step evolution of the carrier profiles during the transient time as the device with double injection layer is switched on by applying a bias voltage of 10.0V. Particularly, in the figures, the formation of charge accumulation nanosheets can be traced during the device turn-on transient time.


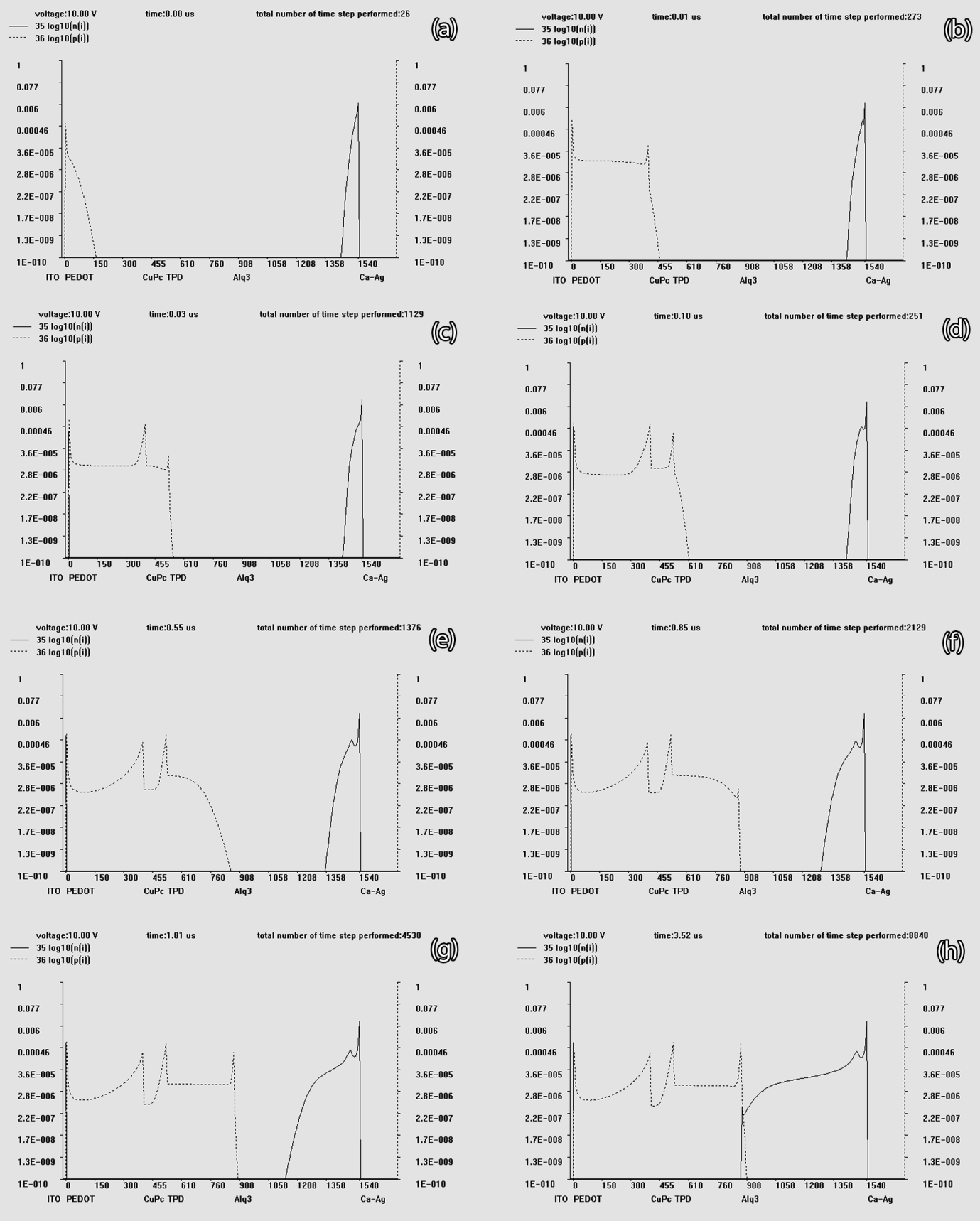


Figure S18. (a) - (.h) Step-by-step evolution of the carrier profiles during the transient time.

Interestingly enough, Figures S19 demonstrate the shift in the molecular energy levels during trun-on transient time. The molecular energy levels are the lowest unoccupied molecular orbital (LUMO) and the highest occupied molecular orbital (HOMO) energy levels. The charge accumulations within the organic layers affect considerably the material energy levels within the device structure. As can be seen, by injection of charge carrier into the organic layers during the device turn-on, the electron and hole molecular energy levels are shifted due to the accumulation of charge carriers among the organic bulk *i.e.* the space charges within the device structure.


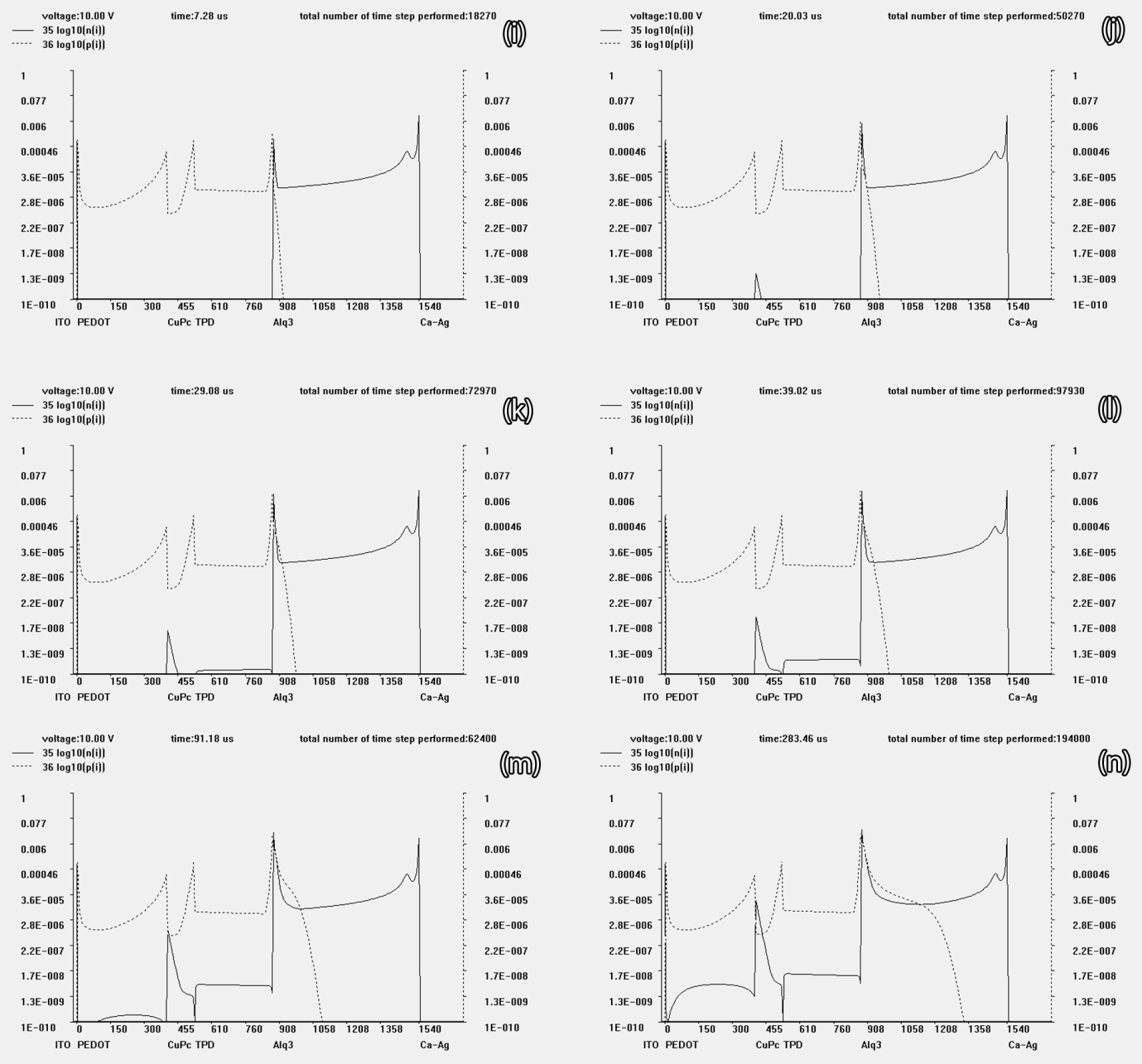


Figure S18 (i) – (n) Step-by-step evolution of the carrier profiles during the transient time.


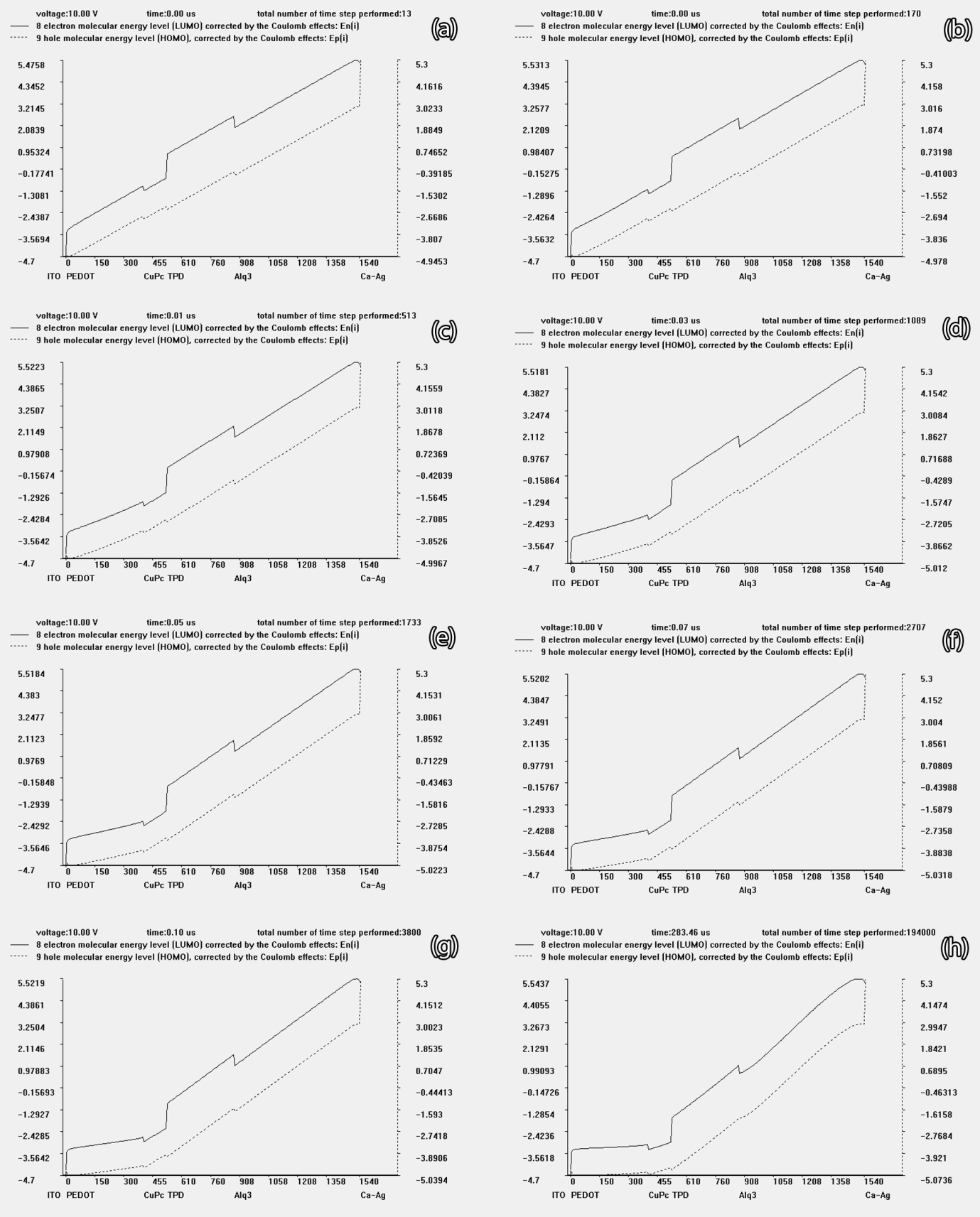


Figure S19 (a) – (h) Electron and hole molecular energy level shift during the transient time.


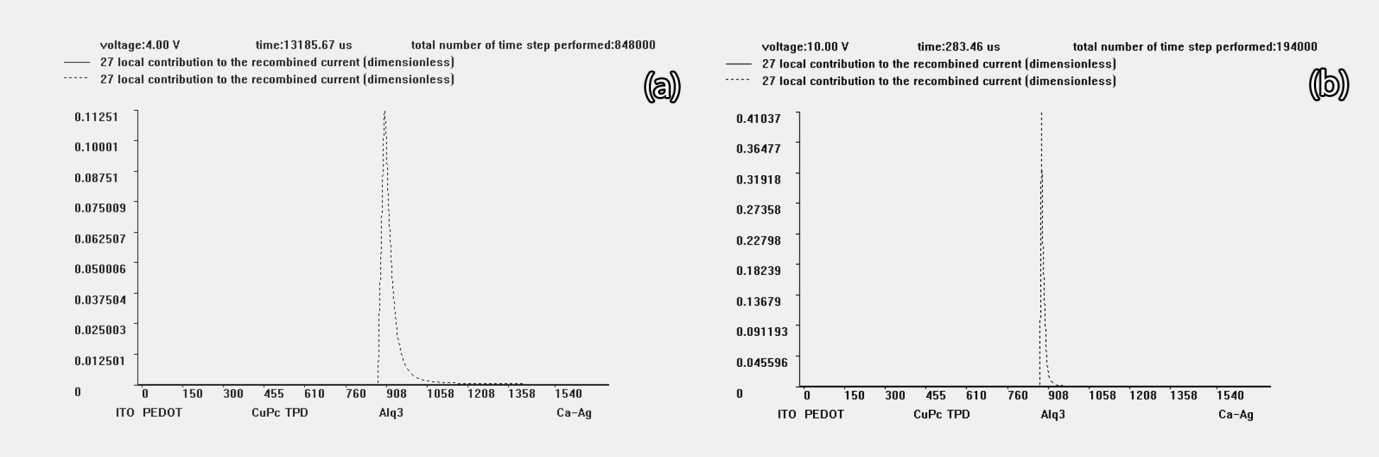


Figure S20.Electron-hole recombination profile for the device with double injection layer, (a) at the bias voltage of 4.0V, and (b) at the bias voltage of 10.0V.

Various microscopic quantities should be calculated during the device simulation, which their evolution can be traced as the time is advanced. For instance, Figure S20 illustrates the electron-hole recombination profile for the device with double injection layer at two bias voltages of 4.0V and 10.0V. Figure S21 demonstrated the tunneling components of the total current density injected into the bulk of the organic device, and Figure S22 illustrates negatively charge carrier and positively charge carrier current components through the organic layers.

As mentioned, the developed numerical code can provide real-time visualization of the microscopic physical quantities which brings valuable insight about the physics of the device operation during transient and steady state conditions. Figures S23 (a) – (f) demonstrates the raw snapshots of the charge carrier profiles and the molecular energy levels at the bias voltage of 10.0V generated by the computer program. These figures are summarizes in the paper as Figure P7. As another example, figures S24 (a) – (f) compare the raw snapshots of the carrier profiles and the molecular energy levels for the three devices as generated in real-time by the computer program. These figures are also presented in the paper as Figure P6.

In conclusion, in the paper and through this supplementary, we presented that device modeling not only can provide deep insight into the understanding of the device performance and its characteristic improvements but also the device simulation can be employed as a comprehensive tool for optimizing the device fabrication procedure. Despite the fact that simulation of a single device might take long time, even few days, and also the fact that we need to simulate many devices, for instance, more than 100 devices have been simulated for this study; nonetheless, the coordinated device simulation jointed with laboratory experiments provides an efficient optimization approach for device fabrication.


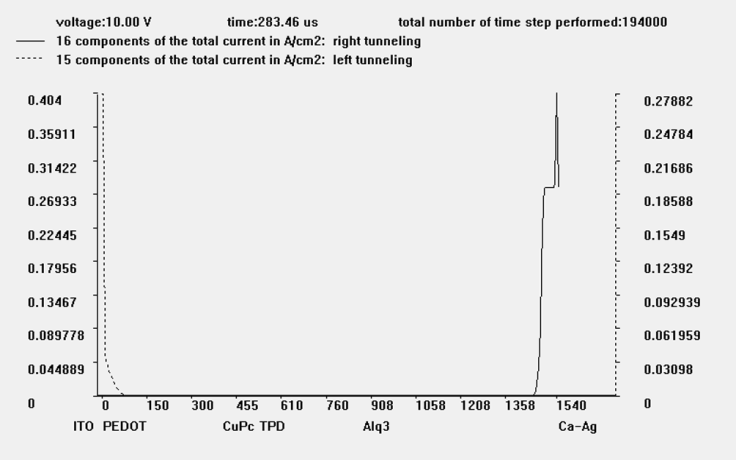


Figure S21. Tunneling components of the total current density injected into the bulk of the organic device.


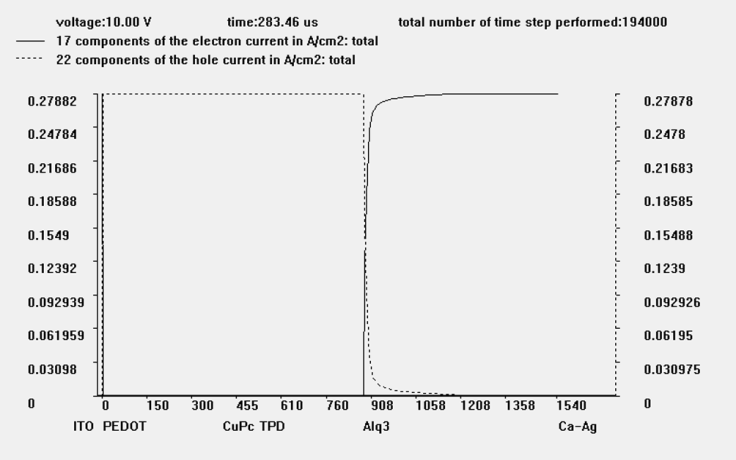


Figure S22. Negatively charge carrier (electrons) and positively charge carrier (holes) current components through the organic layers.


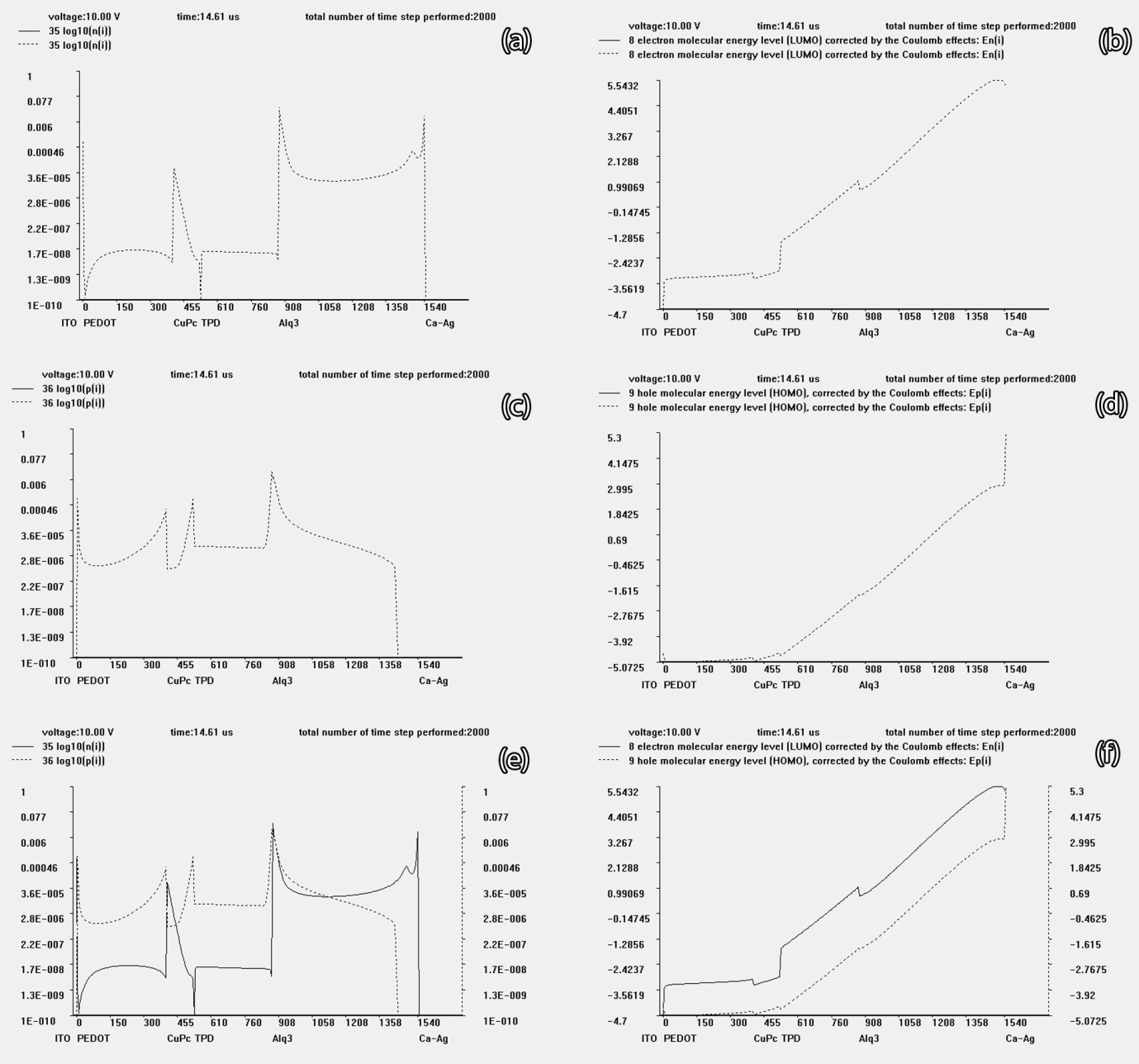


Figure S23. Real-time snapshots of the charge carrier profiles and the molecular energy levels for the device with double injection layer at the bias voltage of 10V as generated by the computer program, in analogy with Figure P7 of the paper.


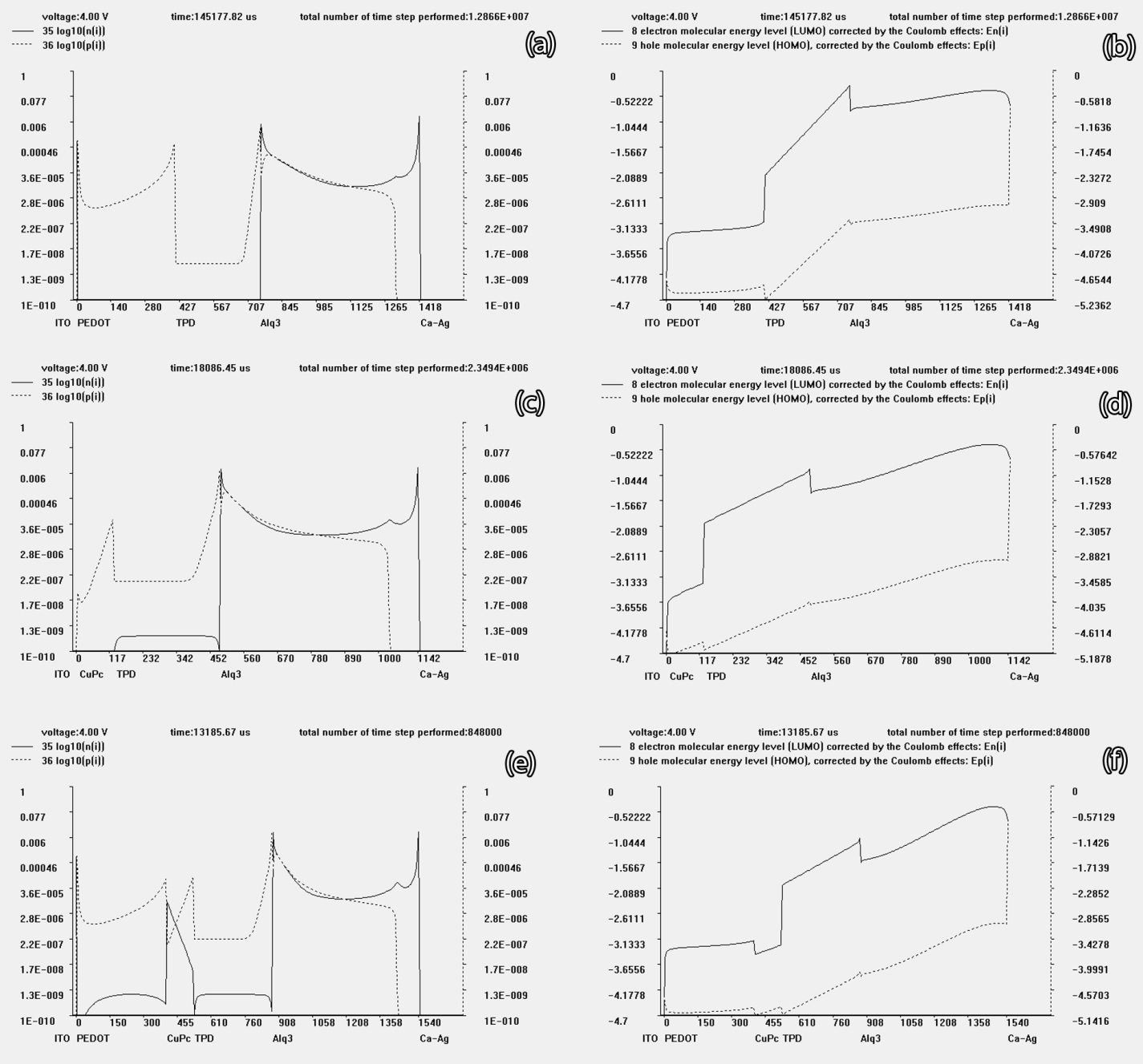


Figure S24. Comparing the real-time snapshots of the carrier profiles and the molecular energy levels for the three devices as generated by the computer program, in analogy with the Figure P6 of the paper.

**Appendix:**

As previously mentioned, device performance is improved according to simulation results by employing PEDOT:PSS and MoO3 together as an organic/inorganic heterointerface injection layer. In the literature, utilization of MoO3 has been investigated together or in combination with PEDOT:PSS. Despite the fact that these structures are not exact replications of the structure investigated here; nonetheless, the similarity in results is particularly noticeable. Here, we review some papers. Referring to Figure S25, Cai and coworkers showed that device performance improves by utilization of ultrathin layer of MoO3 [^13^](#_ENREF_13).


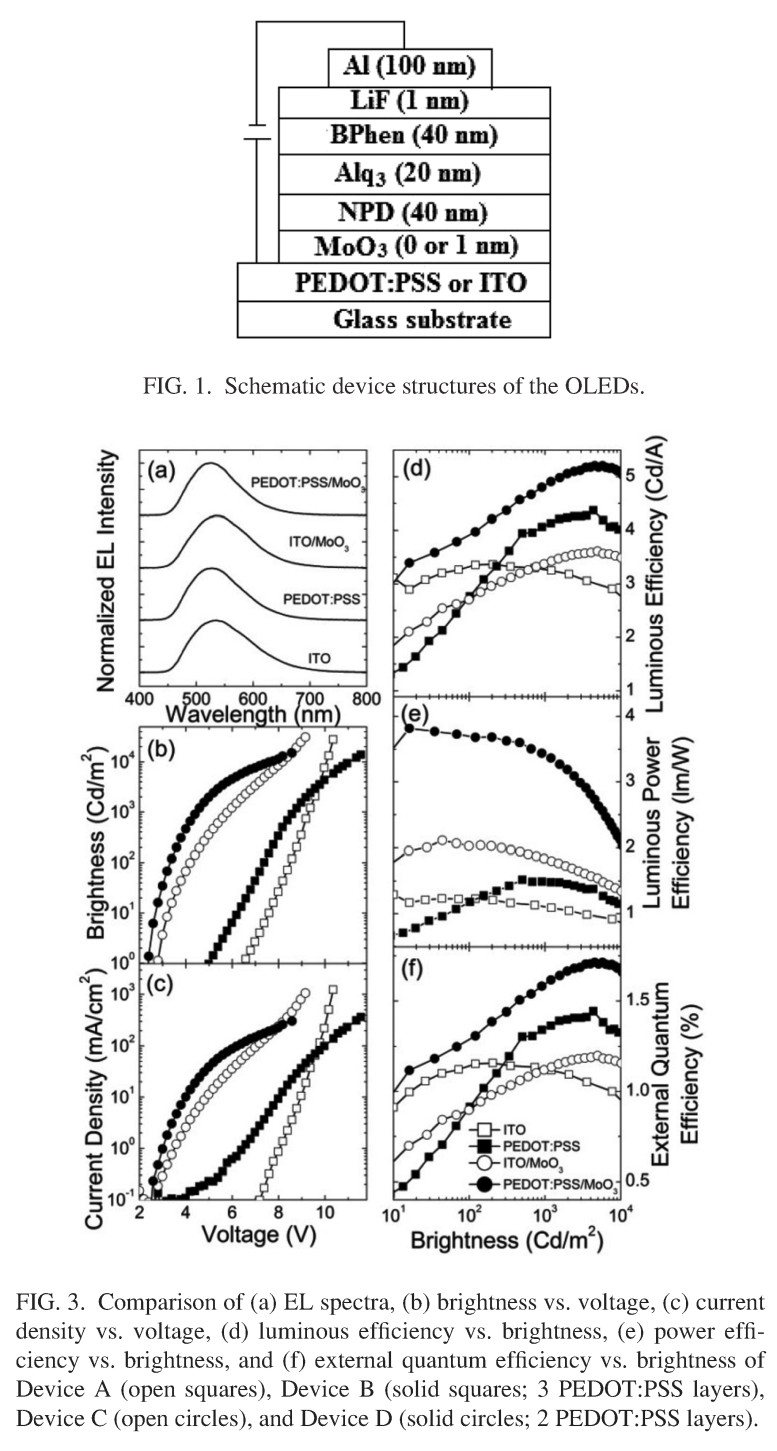


Figure S25. OLEDs employing ITO, PEDOT:PSS, ITO/MoO_3_, and PEDOT:PSS/MoO_3_ as anode and HIL, device structure and comparison of device characteristics. Reprinted from Cai, M., Xiao, T., Liu, R., Chen, Y., Shinar, R., and Shinar, J., Applied Physics Letter, 99, 153303 (2011), with the permission of AIP Publishing[^13^](#_ENREF_13).

According to Figure S26, Zhang *et al.* demonstrated that stacking the materials results in the highest performance for the device[^14^](#_ENREF_14).


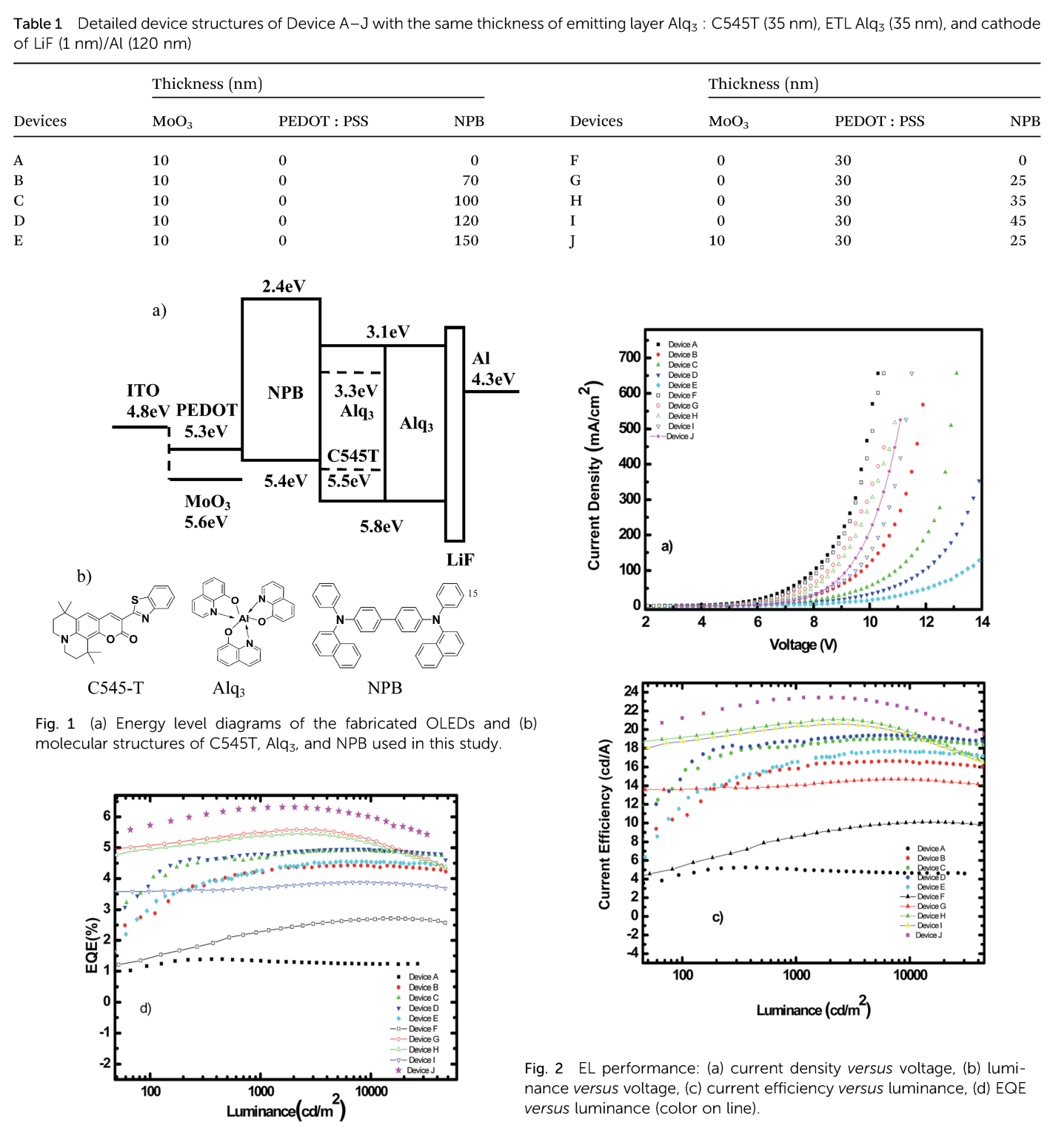


Figure S26. OLEDs employing MoO3, PEDOT:PSS, and MoO_3_/PEDOT:PSS as HIL, device structure and comparison of device characteristics. Reproduced from Ref 14 with permission of The Royal Society of Chemistry[^14^](#_ENREF_14).

Referring to Figure S27, Lee and coworkers presented the device performance improves by employing the hybrid of PEDOT:PSS-MoO_3_ [^15^](#_ENREF_15).


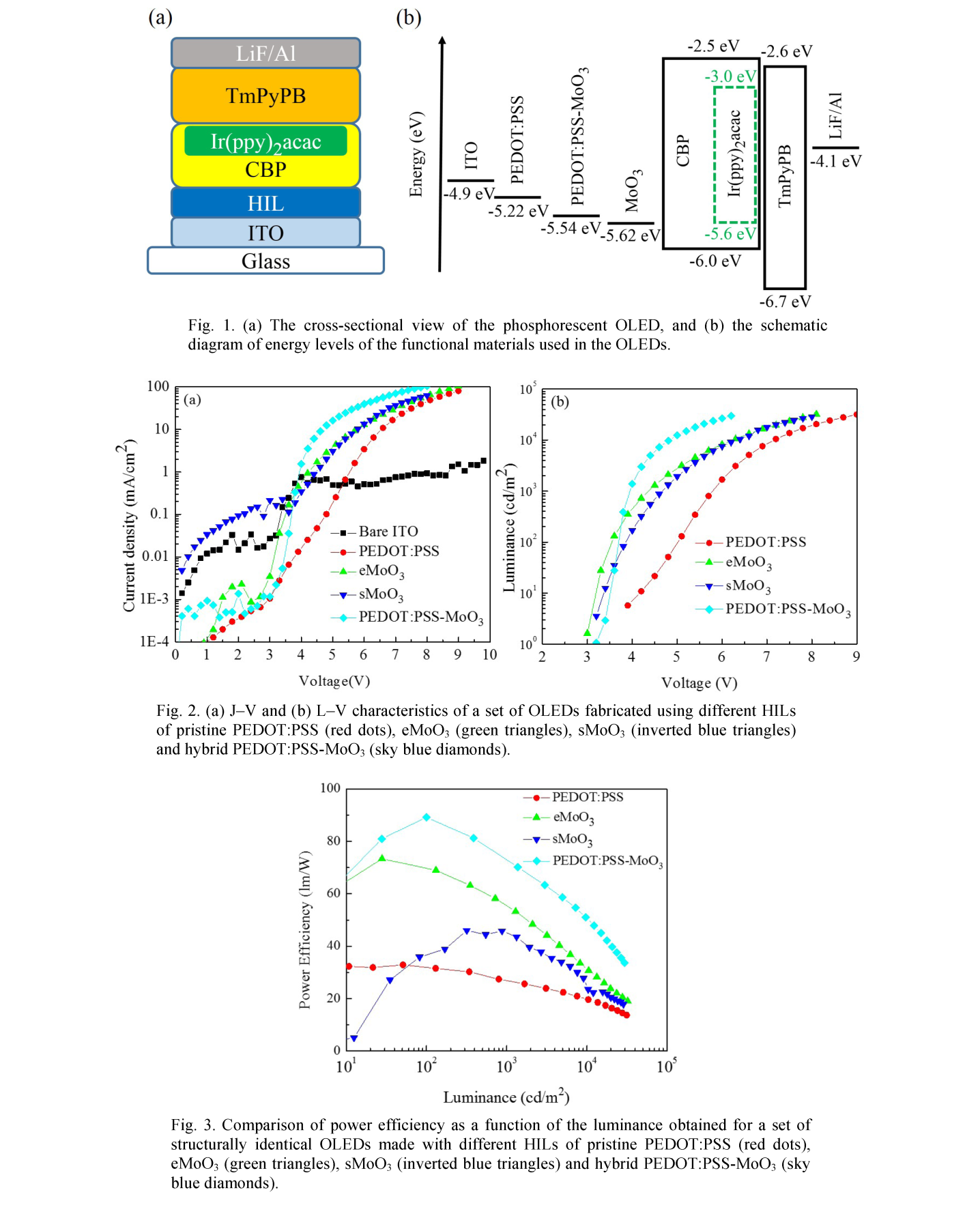


Figure S27. OLEDs employing bare ITO, PEDOT:PSS, eMoO_3_, sMoO3, and PEDOT:PSS/MoO_3_ as HIL, device structure and comparison of device characteristics. Reprinted with permission from Ref 15, The Optical Society[^15^](#_ENREF_15).

Finally, according to Figure S28, Jiang and his colleagues showed the device performance is enhanced when CuPc is replaced by MoO_3_ in device with single injection layer structure[^16^](#_ENREF_16).


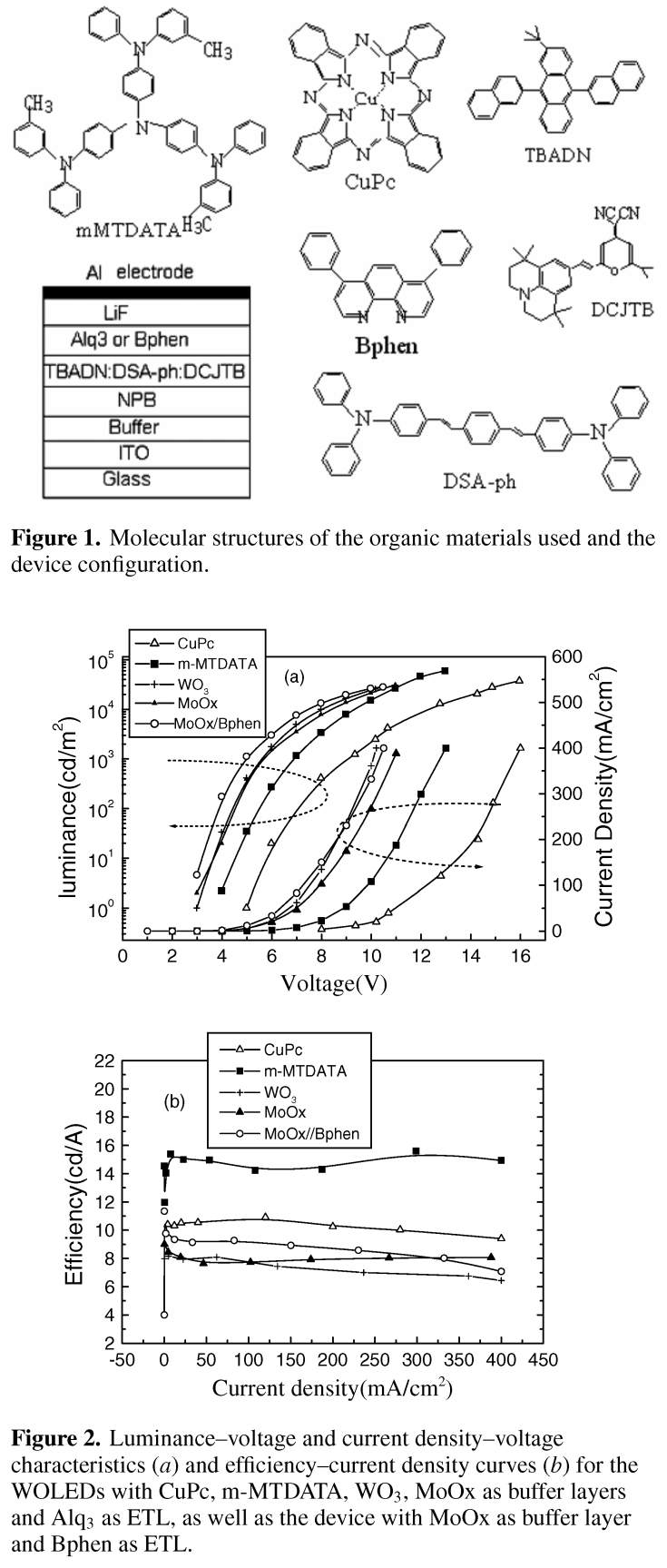


Figure S28. . OLEDs employing CuPc, m-MTDATA, WO_3_, MoO_x_, and MoO_x_ (with Bphen) as HIL. Bphen was used as electron transporting layer, ETL, device structure and comparison of device characteristics. Jiang, X.-Y., Zhang, Z.-L., Cao, J., Khan, M., and Zhu, W.-Q., White OLED with high stability and low driving voltage based on a novel buffer layer MoOx, Journal of Physics D: Applied Physics, 40, 12, 5553, 30 August 2007, © IOP Publishing. Reproduced with permission. All rights reserved[^16^](#_ENREF_16).

1 Fathollahi, M.-R., Boroumand, F. A., Raissi, F. & Sharifi, M.-J. Quantitative characterization of carrier injection across metal–organic interfaces using Bardeen theory. *Org. Electron.* **13**, 905-913 (2012). <http://dx.doi.org/10.1016/j.orgel.2012.01.029>.

2 Zilberberg, K., Meyer, J. & Riedl, T. Solution processed metal-oxides for organic electronic devices. *J. Mater. Chem. C* **1**, 4796-4815 (2013). <http://dx.doi.org/10.1039/C3TC30930D>.

3 Balendhran, S. *et al.* Enhanced charge carrier mobility in two‐dimensional high dielectric molybdenum oxide. *Adv. Mater.* **25**, 109-114 (2013). <http://dx.doi.org/10.1002/adma.201370007>.

4 Masenelli, B., Tutis, E., Bussac, M. & Zuppiroli, L. Numerical model for injection and transport in multilayers OLEDs. *Synth. Met.* **122**, 141-144 (2001). <http://dx.doi.org/10.1016/S0379-6779(00)01350-3>.

5 Tutiš, E., Bussac, M.-N., Masenelli, B., Carrard, M. & Zuppiroli, L. Numerical model for organic light-emitting diodes. *J. Appl. Phys.* **89**, 430-439 (2001). <http://dx.doi.org/10.1063/1.1327286>.

6 Masenelli, B., Berner, D., Bussac, M., Nüesch, F. & Zuppiroli, L. Simulation of charge injection enhancements in organic light-emitting diodes. *Appl. Phys. Lett.* **79**, 4438-4440 (2001). <http://dx.doi.org/10.1063/1.1426683>.

7 Houili, H., Tutiš, E., Lütjens, H., Bussac, M.-N. & Zuppiroli, L. MOLED: Simulation of multilayer organic light emitting diodes. *Comput. Phys. Commun.* **156**, 108-122 (2003). <http://dx.doi.org/10.1016/S0010-4655(03)00435-1>.

8 Tutiŝ, E., Berner, D. & Zuppiroli, L. Internal electric field and charge distribution in multilayer organic light-emitting diodes. *J. Appl. Phys.* **93**, 4594-4602 (2003). <http://dx.doi.org/10.1063/1.1558208>.

9 Berner, D., Houili, H., Leo, W. & Zuppiroli, L. Insights into OLED functioning through coordinated experimental measurements and numerical model simulations. *physica status solidi (a)* **202**, 9-36 (2005). <http://dx.doi.org/10.1002/pssa.200406905>.

10 Fathollahi, M.-R. & Boroumand, F. A. Fabrication and simulation of polyfluorene-based organic light-emitting diodes. *Electrical Engineering (ICEE), 2012 20th Iranian Conference on* **20**, 77; doi:10.1109/IranianCEE.2012.6292327 (2012). <http://dx.doi.org/10.1109/IranianCEE.2012.6292327>.

11 Bussac, M., Michoud, D. & Zuppiroli, L. Electrode injection into conjugated polymers. *Phys. Rev. Lett.* **81**, 1678 (1998). <http://doi.org/10.1103/PhysRevLett.81.1678>.

12 Fathollahi, M.-R., Sharifi, M.-J., Boroumand, F. A., Raissi, F. & Mohajerani, E. Alternative model for injection-limited current into organic solids. *J. Photon. Energy* **5**, 057610-057610 (2015). <http://doi.org/10.1117/1.JPE.5.057610>.

13 Cai, M. *et al.* Indium-tin-oxide-free tris (8-hydroxyquinoline) Al organic light-emitting diodes with 80% enhanced power efficiency. *Appl. Phys. Lett.* **99**, 153303 (2011). <http://dx.doi.org/10.1063/1.3634210>.

14 Zhang, H., Fu, Q., Zeng, W. & Ma, D. High-efficiency fluorescent organic light-emitting diodes with MoO3 and PEDOT: PSS composition film as a hole injection layer. *J. Mater. Chem. C* **2**, 9620-9624 (2014). <http://dx.doi.org/10.1039/C4TC01310G>.

15 Lee, M. H., Choi, W. H. & Zhu, F. Solution-processable organic-inorganic hybrid hole injection layer for high efficiency phosphorescent organic light-emitting diodes. *Opt. Express* **24**, A592-A603 (2016). <http://doi.org/10.1364/OE.24.00A592>.

16 Jiang, X.-Y., Zhang, Z.-L., Cao, J., Khan, M. & Zhu, W.-Q. White OLED with high stability and low driving voltage based on a novel buffer layer MoOx. *J. Phys. D: Appl. Phys.* **40**, 5553 (2007). <http://dx.doi.org/10.1088/0022-3727/40/18/007>.
